# Ferric citrate and apo-transferrin enable erythroblast maturation with β-globin from hemogenic endothelium

**DOI:** 10.1038/s41536-023-00320-4

**Published:** 2023-08-25

**Authors:** Soo-Been Jeon, Hyebin Koh, A-Reum Han, Jieun Kim, Sunghun Lee, Jae-Ho Lee, Seung-Soon Im, Young-sup Yoon, Jong-Hee Lee, Ji Yoon Lee

**Affiliations:** 1 CHA Advanced Research Institute, Bundang CHA Medical Center, CHA University, Seongnam, Kyunggi-do 13488 South Korea; 2 Futuristic Animal Resource & Research Center (FARRC), Korea Research Institute of Bioscience and Biotechnology (KRIBB), Cheongju, Republic of Korea; 3 Department of Functional Genomics, KRIBB School of Bioscience, Korea University of Science and Technology (UST), Daejeon, Republic of Korea; 4 National Primate Research Center (NPRC), Korea Research Institute of Bioscience and Biotechnology (KRIBB), Cheongju, Republic of Korea; 5 Department of Physiology, Keimyung University School of Medicine, Daegu, 42601 Korea; 6 Severance Biomedical Science Institute, Yonsei University College of Medicine, Seoul, Korea; 7 Department of Medicine, Emory University, Atlanta, USA; 8 Department of Biomedical Science, CHA University, Seongnam, Kyunggi-do 13488 South Korea; 9 Present Address: Severance Biomedical Science Institute, Yonsei University College of Medicine, Seoul, Korea

**Keywords:** Embryonic stem cells, Cell growth

## Abstract

Red blood cell (RBC) generation from human pluripotent stem cells (PSCs) offers potential for innovative cell therapy in regenerative medicine as well as developmental studies. Ex vivo erythropoiesis from PSCs is currently limited by the low efficiency of functional RBCs with β-globin expression in culture systems. During induction of β-globin expression, the absence of a physiological microenvironment, such as a bone marrow niche, may impair cell maturation and lineage specification. Here, we describe a simple and reproducible culture system that can be used to generate erythroblasts with β-globin expression. We prepared a two-dimensional defined culture with ferric citrate treatment based on definitive hemogenic endothelium (HE). Floating erythroblasts derived from HE cells were primarily CD45^+^CD71^+^CD235a^+^ cells, and their number increased remarkably upon Fe treatment. Upon maturation, the erythroblasts cultured in the presence of ferric citrate showed high transcriptional levels of β-globin and enrichment of genes associated with heme synthesis and cell cycle regulation, indicating functionality. The rapid maturation of these erythroblasts into RBCs was observed when injected in vivo, suggesting the development of RBCs that were ready to grow. Hence, induction of β-globin expression may be explained by the effects of ferric citrate that promote cell maturation by binding with soluble transferrin and entering the cells.

Taken together, upon treatment with Fe, erythroblasts showed advanced maturity with a high transcription of β-globin. These findings can help devise a stable protocol for the generation of clinically applicable RBCs.

## Introduction

Since their discovery in 1998, human embryonic stem cells (hESCs) have been considered a promising cell source for therapeutic applications in regenerative medicine^1^. The clinical feasibility of human pluripotent stem cells (hPSCs) depends on the controlled differentiation of these cells toward specific lineages with homogeneous transplantable cells and the eventual generation of functional progenies by maturation. Cultured red blood cells (RBCs) from PSCs have recently emerged as potential alternative cell sources in regenerative medicine. Several factors hamper the development of functional RBCs, such as enucleation, cell membrane fragility, and low β-globin expression in erythrocytes. The development of functional RBCs has been constantly examined under in vitro conditions^2–6^. By previous reports, OP9 feeder cells are used as an indispensable factor to induce β-globin. However, the clinical application of therapeutic cells would require at least feeder- and xenogeneic-free system. Our protocol can be accomplished the induction of β-globin by simple method accompanying with Fe treatment. Achieving β-globin expression after enucleation is the final step in RBC maturation^7,8^. Erythropoiesis of mammalian cells is a complex process during which mature RBCs are generated from hematopoietic stem cells (HSCs) in the postnatal stage. Similar to the postnatal stage process, embryo-derived erythropoiesis consists of two phases, namely, primitive erythropoiesis originating in the yolk sac with nucleated erythroblasts expressing embryonic (ζ_2_ɛ_2_) hemoglobin and definitive erythropoiesis originating in the fetal liver with enucleated erythrocytes expressing fetal (α_2_γ_2_) and adult hemoglobin (α_2_β_2_)^9–11^. Most mature RBCs gradually transition from α_2_γ_2_ to α_2_β_2_ hemoglobin after birth, demonstrating equilibrium without hemolysis^12,13^. Unlike mice which undergo RBC maturation by a switching process in the fetal liver, this switch to β-globin from γ-globin is unique to humans^14,15^, and results in a distinct differentiation process during RBC maturation. Therefore, diverse applications, including liver- or thymic stromal cell co-culture systems, have been developed to obtain mature RBCs with β-globin expression under in vitro culture conditions, with hematopoietic cytokines added for specification^16–18^. Owing to the scarcity of human yolk sac and aorta gonad mesonephros, understanding blood hierarchy via ontogeny using hPSCs is indispensable for obtaining specific hematopoietic lineage cells, such as RBCs, for application in clinical trials. Since the first examination of erythroid cell maturation in a dish, functional hematopoietic lineage cells, including HSCs, RBCs, and T lymphoid lineage cells, have been obtained from definitive hemogenic endothelial cells, which are the precursors to human embryonic HSCs^19,20^. Primitive erythroblasts and erythro-myeloid progenitors (EMP) do not show β-globin expression, whereas HSC-derived erythroblasts from definitive hemogenic endothelium (HE) produce mature RBCs expressing β-globin^21–23^. Irrespective of the origin of mature RBCs, to date, consensus dictates that erythroid- and megakaryocyte-committed cells generated from HSCs with early hierarchy depend on definitive HE. These findings prompted us to investigate whether hPSC-derived definitive HE can be used to generate functional erythrocytes with β-globin expression under defined culture conditions. Definitive CD34^+^CXCR4^-^CD73^-^ HE cells, which are essential for inducing erythropoiesis, can potentially differentiate into hematopoietic and endothelial lineage cells^11^. In this study, we first observed differentiation of functional HE cells and successfully developed RBCs with β-globin expression under optimized culture conditions using ferric citrate. Iron is essential for the proliferation of erythroblasts as iron is important for hemoglobin production. However, the effect of ferric citrate on the promotion of the expression of β-globin in erythroblasts has not been elucidated. The cellular and molecular interactions involved in blood cell development can be examined based on two core experimental pillars: prospective cell purification and clonal assays, such as single-cell RNA sequencing. Thus, we developed a stromal-independent culture system that can effectively generate a large number of erythroblasts (basophilic, polychromatophilic, and orthochromatic) from hPSC-derived CD34^+^definitive HE under defined conditions. Subsequently, single-cell RNA sequencing was performed to assess β-globin expression in response to ferric citrate.

Together, these approaches that relied on mimicking ontogenetic processes developed RBCs expressing β-globin from definitive HE. The development of erythroblasts was facilitated by Fe treatment. The present study may provide a platform for defining the academic inquiry in developmental biology as well as for the development of clinical translation strategies in regenerative medicine.

## Results

### PSC-derived erythroblasts were successfully produced by ferric citrate

**Fig. 1:**
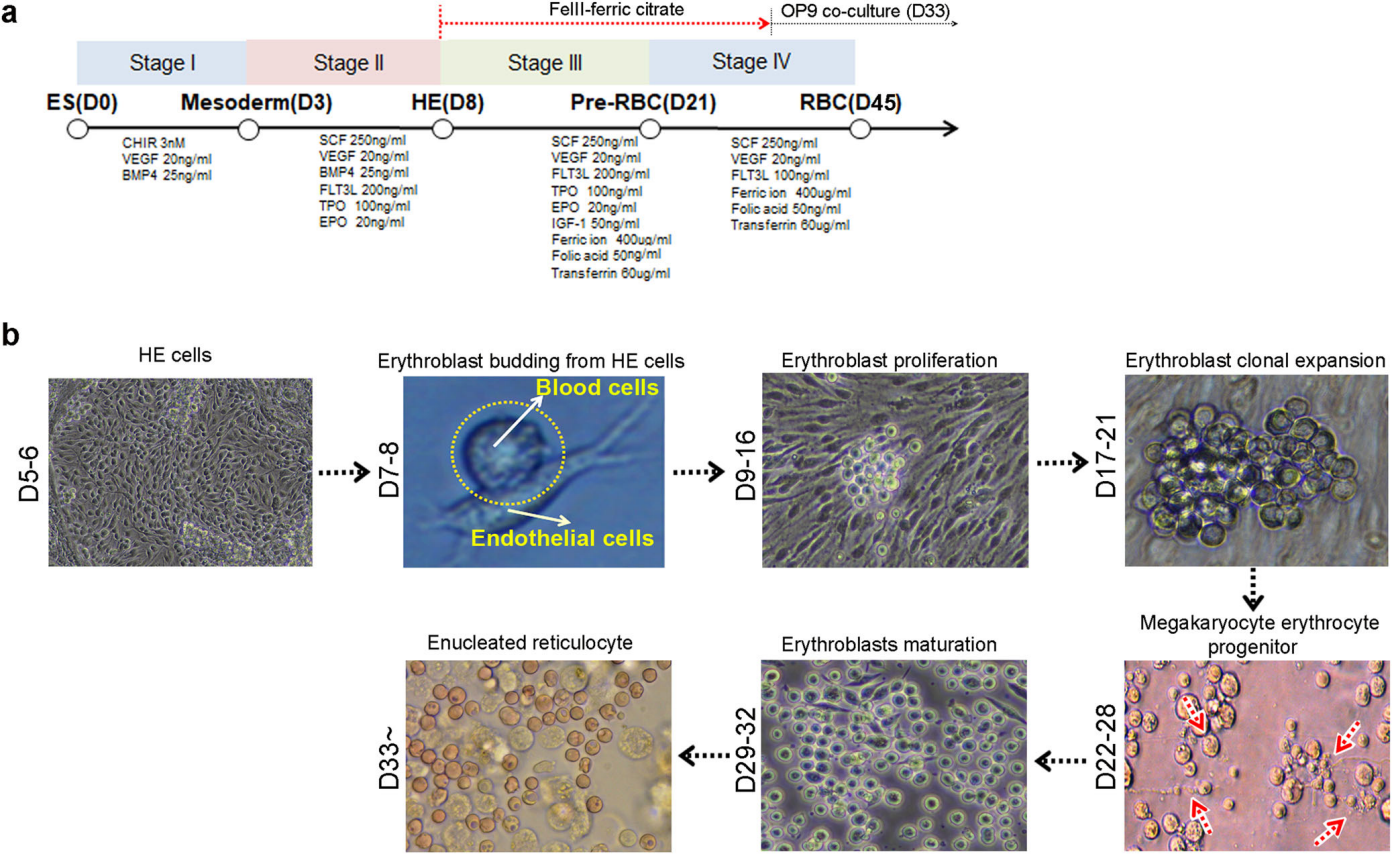
Erythroblasts generated from PSCs and their differentiation in defined culture conditions after addition of ferric citrate. **a** Schema for generating erythroblasts from PSCs *via* CD34 sorted HE. **b** After erythrocyte generation, morphological changes indicate that HE developed into erythroid lineage cells via erythroblasts. Expanded erythroblasts were continuously proliferated by ferric citrate. Definitive hematopoiesis involving megakaryocyte erythrocyte progenitors was observed, and matured erythroblasts were generated till day 32. To obtain mature erythroid lineage cells, OP9 stromal cells were co-cultured, and enucleated erythrocytes were detected on OP9 cells.

To generate functional erythrocytes from human PSCs, we developed a protocol in which cells were cultured for 40 days in a media supplemented with specific cytokines, as shown in Fig. 1a. HE acts as a platform for erythroblast development. Using this protocol, the development of HE was successfully induced from days 6–8 via a process known as endothelial-to-hematopoietic transition (EHT)^24,25^. Based on HE, budding erythroblasts were primarily produced via primitive and definitive hematopoiesis processes followed by clonal expansion (Fig. 1b). To test the basal medium that was optimized for HE production, two media, namely, Apel 2 and Stemline II, were evaluated for the development of HE on day 5. The percentages of markers, including CD34, CDH5, and KDR for HE, were higher in the Apel 2 medium (CD34, 29.4%, CDH5, 22.9%, KDR, 53.2%, CD34^+^KDR^+^, 21.7%, CD34^+^CDH5^+^, 26.0%) compared to those in the Stemline II medium (CD34, 6.0%, CDH5, 4.4%, KDR, 29.5%, CD34^+^KDR^+^, 3.8%, CD34^+^CDH5^+^, 3.5%). No difference were observed between two media with respect to the percentage of CD34^+^CXCR4^-^CD73^-^ definitive HE cells (Apel 2, 94.2%, Stemline II, 91.6%) (Supplementary Fig. 1a). HE has the bipotential capability to differentiate into endothelial cells and hematopoietic lineage cells. Therefore, we tested the tube formation to confirm endothelial properties of CD34^+^ HE. Supplementary Fig. 1b showed that the CD34^+^ HE cells in EGM-2 medium produced generated tube formation (Supplementary Fig. 1b). To effectively obtain the erythroblasts, we used Apel 2 medium to generate erythroblasts. During definitive hematopoiesis, megakaryocyte erythrocyte progenitors were observed in ~21 days. Erythroblasts matured simultaneously into orthochromatic erythroblasts under in vitro defined culture conditions until 32 days. Heterogeneous erythroblasts, including basophilic, polychromatophilic, and partly orthochromatic erythroblasts developed continuously, and most cells were identified as polychromatophilic and orthochromatic erythroblasts on day 32 after differentiation (Fig. 1b, till D29-32). To complete enucleation, OP9 stromal cells were applied to the culture system for 12 days, and small-sized erythrocytes or enucleated RBCs were detected upon morphological analyses within 40 days (Fig. 1b, after D33).

### PSC-derived erythroblasts cultured in a two-dimensional culture system were typically generated by ferric citrate treatment but completion of cell enucleation was not observed

**Fig. 2:**
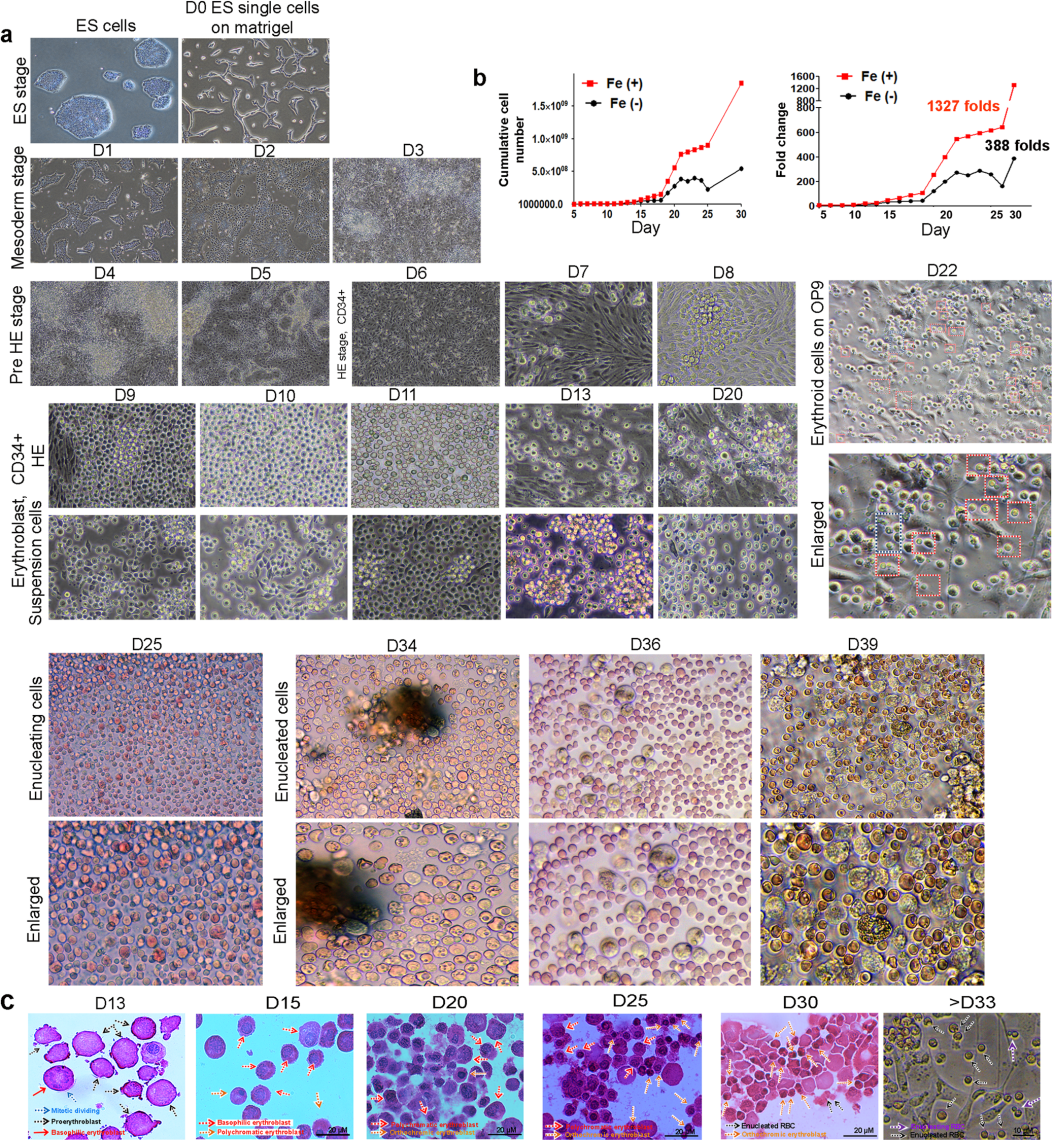
Erythropoiesis was successfully observed under defined culture conditions along with a high proliferation of erythroblasts by ex vivo ferric citrate treatment. **a** Representative morphology of PSC-derived erythroid lineage cells according to specific cytokines. Magnification ×10, ×20, and ×40. Traditional CHA-52 ES single cells were plated on Matrigel until the development of HE via mesodermal lineages. On day 5, CD34^+^ HE was sorted and further cultured, resulting in erythroid lineage cells. The number of floating cells increased remarkably by Fe treatment until day 32. HE cells continuously generated erythroblasts, and megakaryocyte erythrocyte progenitors emerged on days 21–25. Red arrows indicate megakaryocyte erythrocyte progenitors. Subsequently, heterogeneous erythroblasts including basophilic, polychromatophilic, and orthochromatic erythroblasts were obtained from hPSC-derived CD34^+^ definitive HE under defined conditions. Upon co-culturing with the OP9 cells, the number of erythroblasts rapidly decreased, with their cell sizes below 10 µM. The ribosome in the cytoplasm was first excluded, and then the nucleus was next expelled from the erythroblasts, resulting in the complete development of erythrocytes. The blue box shows enucleating cells, and the red boxes show enucleated cells. However, enucleated RBCs were rarely detected on day 33 and stably generated after day 36. **b** The cumulative numbers of erythroblasts were remarkably increased by ~1327-fold after ferric citrate treatment compared to that observed with no treatment. **c** Wright-Giemsa staining clearly showed erythropoiesis procedure under in vitro conditions. Proerythroblasts were mainly detected in the dish on day 13. Blue arrow indicates proerythroblasts undergoing mitotic division. Black arrows show traditional proerythroblasts. Basophilic erythroblasts (red arrows) and polychromatophilic erythroblasts were observed on day 15. These cells with decreased size gradually developed into polychromatophilic erythroblasts (red arrows) and orthochromatic erythroblasts (black arrows) from days 20 to 25. The sizes of these cells reduced rapidly with condensate nucleus, and the basophilic color gradually disappeared. Before co-culturing with OP9 cells, most cells were identified as orthochromatic erythroblasts (orange arrows), and enucleated erythrocytes (black arrows) were occasionally detected on day 30. Upon co-culturing with OP9 cells, the number of enucleated erythrocytes remarkably increased after day 33. Orange arrows show enucleating cells, and black arrows indicate enucleated erythrocytes. Scale bar, 20 µM.

First, we sought to determine whether erythroblasts were morphologically developed via CD34^+^ HE cells and the development stage of erythroblasts that could be observed with a two-dimensional culture system. To this end, starting with 200,000 PSCs, cell differentiation was initiated depending on the Apel 2 medium on a matrigel-coated culture dish. The PSCs developed into mesodermal lineages on day 3 after differentiation and formed HE cells, which comprise bi-potential progenitors that can develop into both hematopoietic lineage cells and endothelial cells on day six. To obtain purified HE cells, MACS sorting was applied with CD34^+^ beads on day 5 to enrich HE (Fig. 2a, D6). Budding of early erythroblasts from stable HE was observed, and clonal expansion of erythroblasts was detected during differentiation (Fig. 2a, D7–8). The number of suspended cells, namely erythroblasts that floated from adherent HE cells, gradually increased upon treatment with ferric citrate. A total of 1,400,000 cells were harvested from the supernatant of one six-well plate, cells were continuously cultured till day 30. We found that cumulated cell number (left) and fold change (right) highly increased in Fe treated group (non-Fe, 388-folds, Fe, 1327-folds) (Fig. 2b). This period permitted diversification of erythroblasts, such as basophilic, polychromatophilic, and orthochromatic erythroblasts in a two-dimensional culture system (Fig. 2a, D9–28). The size of the cells gradually decreased from 12–20 µM to 7–10 µM, suggesting the maturation of erythroblasts. Meanwhile, adherent HE cells successively produced erythroblasts up to day 25. Subsequently, a gradual reduction in cell production was observed. Megakaryocyte erythrocyte progenitor and megakaryoblasts (MK-I) were observed following differentiation after day 21 (Fig. 2a, D21–25). These cells further developed into MK-II and MK-III, leading to platelet shedding on day 25 (data not shown). This implies that a defined culture system allows definitive hematopoiesis^10^. Many trials have examined effective enucleation, including hematopoietic complex formation, HDACi, and a co-culture system^26,27^. Consistent with the findings of these studies, we observed that erythroblasts were grown and developed into erythrocytes. In the two-dimensional culture system, the morphology and cell number of orthochromatic erythroblasts were remarkably increased by ferric citrate treatment, and further maturation into erythrocytes was observed upon addition of OP9 stromal cells. However, the two-dimensional culture system was unable to fully induce enucleated erythrocytes (Fig. 2a D33–39). Subsequently, to test if cell maturation occurred according to differentiation, Wright-Giemsa staining using erythroblasts was performed for each differentiation day. Up to 13 days, proerythroblasts with a nucleus showing a fine chromatin pattern were primarily generated from HE, and basophilic erythroblasts with a round nucleus and basophilic cytoplasm were also subsequently differentiated on day 15. The size of most cells is smaller than that of proerythroblasts. These cells gradually decrease their size depending on development, resulting in the generation of polychromatophilic erythroblasts on day 20. Early orthochromatic erythroblasts, which were emerging on day 25, showed condensed nucleus and pale cytoplasm color. These cells initiated ribosome extrusion of cytoplasm, and the size of cells was reduced to less than 10 µM on day 30. Because many enucleated erythrocytes and pyrenocytes were washed away during preparation for staining after day 33, enucleating erythroblasts and enucleated cells were observed in bright fields instead of analysis via Wright-Giemsa staining. Mitotic division was observed in cells after the development of orthochromatic erythroblasts (Fig. 2c).

### Markers of erythroblasts were highly expressed in ferric citrate-treated group with a strong capacity for CFU-E formation

**Fig. 3:**
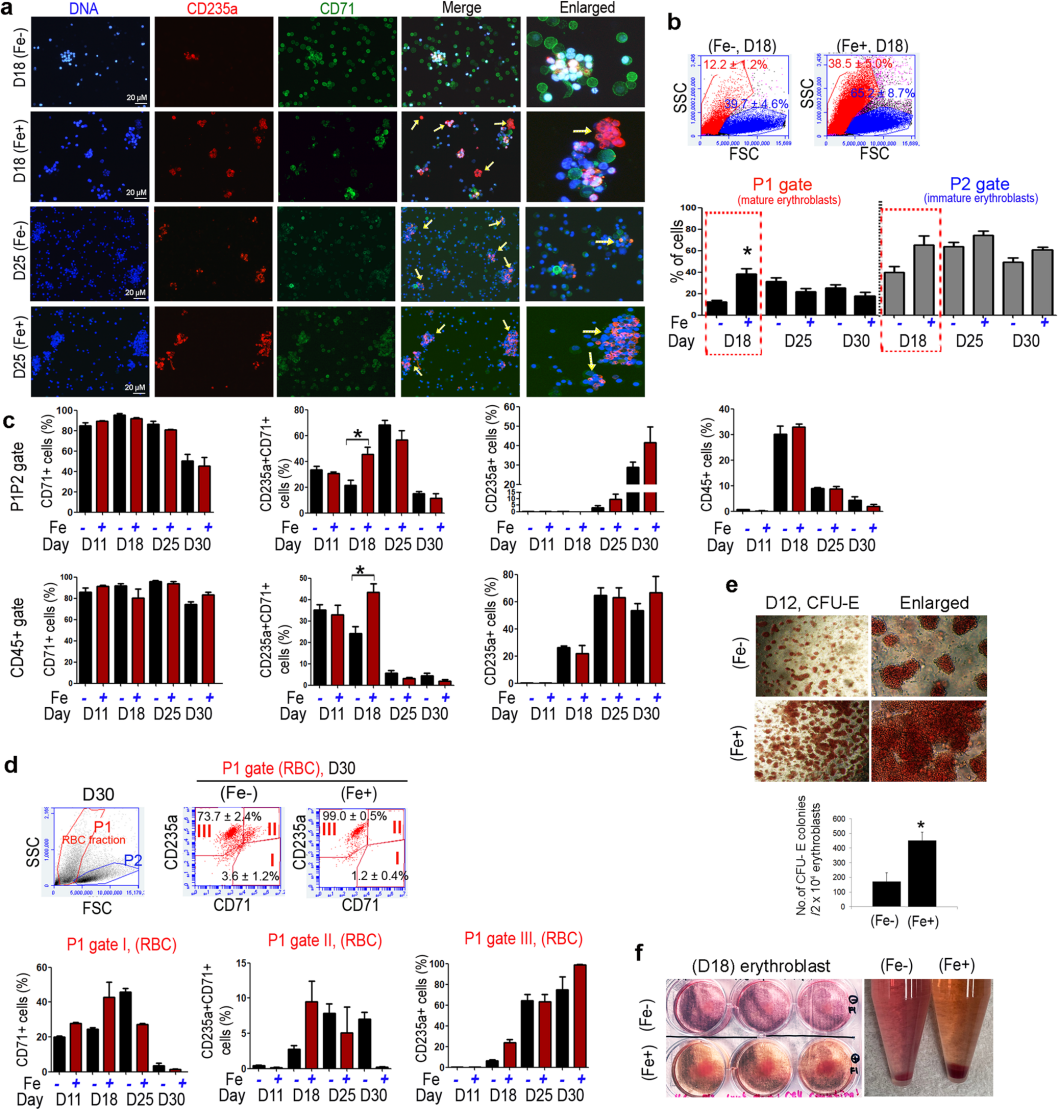
Markers were highly expressed in the ferric citrate-treated group. **a** Immunocytochemistry data showed that the intensity of the CD71 progenitors decreased gradually, and the number of CD235a cells increased significantly with strong intensity. Yellow arrows indicate CD235a^+^ erythroblasts. Images obtained from at least two independent experiments for each cell are shown. CD235a (red), CD71 (green), and DAPI (blue) are clearly demarcated in erythroblasts. Scale bar = 20 µM. **b** Flow cytometry showed that the cell population was divided into two fractions based on the cell sizes during differentiation. The number of both populations, mature-(P1) and immature-(P2) erythroblasts, was highly increased by Fe treatment. *n* = 3–7 per group. *P < 0.05, Mann–Whitney U test. **c** The percentage of CD71^+^, CD71^+^CD235a^+^, CD235a^+^ erythroblasts in whole cells and CD45^+^ immature population is demonstrated based on days and Fe treatment. *n* = 3–8 per group. *P < 0.05, Mann–Whitney U. **d** The percentage of CD71^+^, CD71^+^CD235a^+^, CD235a^+^ erythroblasts in mature population (P1) according to Fe treatment. CD235a matured erythroblasts were primarily enriched with a low number of CD71 immature cells in the P1 fraction. *n* = 3–6 per group. All data are derived from at least three independent experiments. **e** The ability of early erythroblasts to form CFU-E colonies after the addition of Fe was examined. Data are represented as means ± SEM derived from at least two independent experiments. *n* = 6–7 per group. *P < 0.05. **f** Red-colored erythroblasts were observed on day 18, implying hemoglobin synthesis was observed with a high cumulative number of cells in the Fe-treated group.

To examine whether differentiated erythroblasts expressed the markers for erythroblasts including CD45, CD71, and CD235a (glycophorin A), we performed immunocytochemistry and flow cytometry analyses. Regardless of Fe treatment, the CD71 pan erythroblast marker was continuously expressed in erythroblast lineages. On day 18, mature CD235a, a maturation marker of erythroblasts, was qualitatively detected with low levels of CD71^+^ progenitor cells in the Fe-treated group compared to that in the non-treated group. Meanwhile, the overall number of CD71^+^ progenitor cells decreased remarkably on day 25 with an increase in CD235a cells. This qualitative imaging suggests maturation of erythroblasts (Fig. 3a). Cell scattering plots also showed that the number of differentiated cells was significantly increased in a fraction of mature erythroblasts (P1 gate, untreated, 12.2 ± 1.2%, Fe, 38.5 ± 5.0%) as well as in erythroblasts (P2 gate, non-Fe, 39.7 ± 4.6%, Fe, 65.2 ± 8.7%) on day 18. On day 25 and 30, an increase in the number of immature erythroblasts was observed in the Fe-treated group, suggesting the effects of Fe in the proliferation of the erythroblasts (Fig. 3b). CD45^+^ immature erythroblasts expressing CD71 were still undergoing mitotic division; even their intensity gradually dimmed compared to that of erythroblasts at day 18. Subsequently, as the cell size of erythroblasts varied significantly from 6–7 µM to 20 µM depending on their development, we analyzed the percentage of markers in all cells (P1 and P2 gate), except the debris fraction. CD71 was continuously expressed during differentiation, and CD235a expression was gradually increased, as observed with the Fe-treated group. The number of cells co-expressing CD71 and CD235a was significantly increased in the Fe-treated group on day 18 (non-Fe, 21.5 ± 3.7%, Fe, 45.4 ± 5.7%). In CD45^+^ immature erythroblasts, the percentages of CD71^+^CD235a^+^ erythroblasts were significantly increased by Fe treatment on day 18 (non-Fe, 24.1 ± 3.2%, Fe, 43.5 ± 3.8%) (Fig. 3c), suggesting the effects of ferric citrate in increasing the number of CD71^+^CD235a^+^ erythroblasts. Next, our data showed that the expression of pan hematopoietic cell marker CD45 gradually decreased after 25 days according to the development of erythroid lineages, whereas the number of CD235a^+^CD45^+^ cells rapidly increased after day 25, implying cell maturation. On day 30, the fraction comprising RBCs with small sizes, which may mature into erythroid lineages and is represented by CD45^-^ phenotype, was clearly detected in the cell scattering plot. We specifically gated the P1 gate at the site of RBC concentration to examine the mature pattern of erythroid lineage cells. FACS data showed that CD235a^+^ cells were gradually increased and CD235a^+^CD71^+^ cells were decreased in the RBC fraction after Fe treatment (only in CD235a^+^ cells marked as III; non-Fe, 73.7 ± 2.4%, Fe, 99.0 ± 0.5%, in CD235a^+^CD71^+^ cells marked as II; non-Fe, 7.0 ± 0.9%, Fe, 0.2 ± 0.0%). On day 30, the erythroid progenitor only in CD71^+^ cells marked as I were also rapidly decreased regardless of the Fe treatment (non-Fe, 3.6 ± 1.2%, Fe, 1.2 ± 0.4%) (Fig. 3d). Meanwhile, we found that the P2 gate had a higher number of CD235a^+^CD71^+^ cells compared to the P1 gate, suggesting that immature cells were enriched in the P2 gate (Supplementary Fig. 2). The findings suggest RBC maturation under defined culture conditions by Fe treatment. To investigate the potential of early erythroblasts in colony formation, a CFU assay was performed using differentiated erythroblasts. If differentiated cells could be characterized as ancestors of RBCs, CFU-E (erythroblasts) would be observed within several days. Early erythroblast cells lead to CFU-E formation regardless of the Fe treatment. However, the frequency of colonies was significantly higher by ~2.6-fold in the Fe-treated group compared to the untreated group (Fig. 3e). These cells matured in dishes under in vitro conditions with a red color, suggesting hemoglobin synthesis. Hemoglobin synthesis was accompanied by a high proliferation of erythroblasts in the Fe-treated group (Fig. 3f).

### Fetal- and β-globin switching occurs in parallel with OP9 co-cultured erythroblasts

**Fig. 4:**
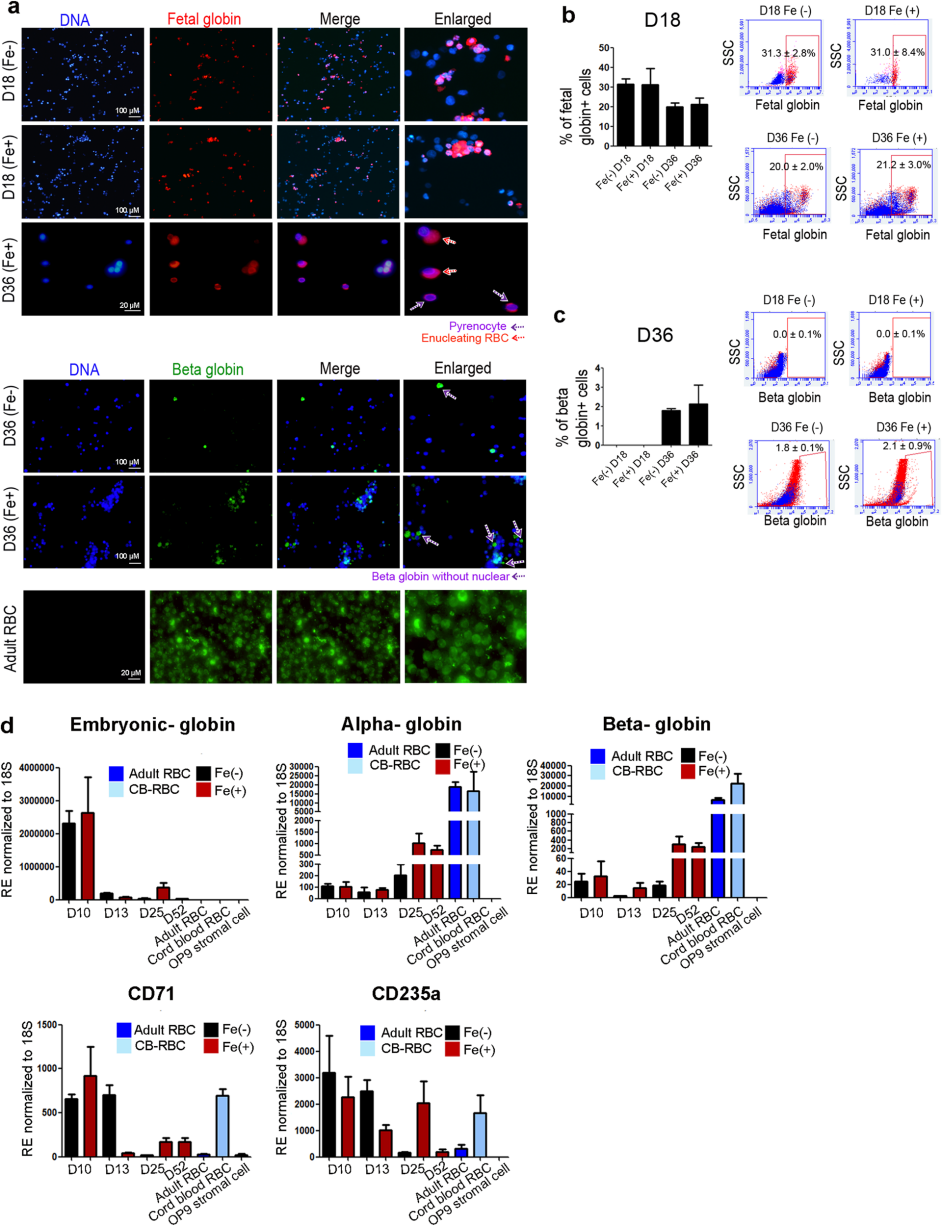
The expression of fetal- and β-globin upon co-culturing with OP9 cells was observed in the Fe-treated group on day 36. **a** Immunocytochemistry data clearly showed that fetal globin was detected in early erythroblasts, regardless of the addition of Fe. Fetal globin expression was detected in erythroblasts with a nucleus and enucleating erythroblasts. Purple arrows depict pyrenocytes, and red arrows indicate enucleating erythroblasts. A high expression of β-globin was detected in the Fe-treated group compared to that in the non-Fe-treated group. Purple arrows depict β-globin in enucleated erythroblasts. Scale bar = 20 µM and 100 µM. **b** Flow cytometric analysis of fetal globin cells showed no significant difference between groups. Data are represented as means ± SEM derived from two independent experiments. *n* = 2–3 per group. **c** Flow cytometric analysis of fetal globin cells showed no significant difference between groups. Data are represented as means ± SEM derived from three independent experiments. *n* = 3 per group. CB-RBC and adult PB-RBC were used as alternate positive controls. **d** The results of qRT-PCR revealed a significantly high mRNA expression corresponding to alpha globin and β-globin in differentiated cells in the Fe-treated group. Data are represented as means ± SEM derived from three independent experiments performed with duplicates. *n* = 6–18 per group.

To examine whether globin was expressed in developed erythroblasts, we performed immunocytochemistry and FACS analysis using differentiated erythroblasts. Unlike other protein movements, globin expression and switching patterns in enucleated erythroid cells are not influenced by cytokines and development days. Hence, RBCs may not be allowed to mature in a two-dimensional culture supplemented with various cytokines. Until day 36, most floating erythroblasts having a nucleus were positive for γ-globin but not for β-globin (Fig. 4a, upper panel). In contrast, the erythroblasts observed with the OP9 stromal cells was positive for γ-, α-, and β-globin; however, the expression of β-globin was not as strong as that of the adult fetal-globin (Fig. 4a, low panel). Some erythroblasts with or without a nucleus showed β-globin expression. Erythroblast transition from γ-globin to β-globin was observed on day 36 in OP9 cells, indicating maturation of erythrocytes. Regardless of the Fe treatment, the expression of fetal globin was consistently sustained at approximately 30% and 20% on days 18 and 36, respectively, indicating a decreased expression (on day 18, non-Fe, 31.3 ± 2.8%, Fe, 31.0 ± 8.4%; on day 36, non-Fe, 20.0 ± 2.0%, Fe, 21.2 ± 3.0%) (Fig. 4b). Meanwhile, no fetal globin expression and approximately 14.8% of fetal globin expression were detected in cord blood (CB)-derived RBCs and peripheral blood cells, respectively (data not shown). The expression of β-globin in erythroblasts was initiated upon the addition of OP9 stromal cells after differentiation on day 36 (non-Fe, 1.8 ± 0.1%, Fe, 2.1 ± 0.9%) (Fig. 4c). Although a significant difference was not detected both groups, the Fe-treated group showed a trend of increased expression compared to the non-Fe treated group. In adults, approximately 14.8% and 99.9% of β-globin expression was observed in CB-derived RBCs and peripheral blood cells, respectively (data not shown). Next, quantitative real-time PCR analysis of globin genes in floating erythroblasts was performed to confirm the globin switch at the mRNA level. The cDNA of differentiated erythroblasts on days 10, 13, and 25 in Fe- and non-Fe-treated groups and that of erythroid cells in the Fe-treated groups on day 52 was obtained. Alternative positive controls were adult peripheral RBCs and CB-RBCs, whereas OP9 stromal cells were used as the negative control. The embryonic (ε, epsilon) globin expression peaked at day 10 and then gradually decreased. Meanwhile, adult globin (α, alpha and β, beta) was increased considerably in a day-dependent manner. The alpha-globin constantly showed high expression from the sixth week of gestation to the postnatal stage in development. The synthesis of adult β globin was completed and sustained at 18 weeks after birth, implying that acquirement of β-globin is required to take long periods after migration to BM. Notably, the transcript level of β-globin showed an ~8.8-fold increase in the Fe-treated group and an 11.8-fold increase in the non-Fe-treated group after day 25 compared to its expression on day 10 (Fig. 4d). PCR data showed that the transcript levels of the globin family, including α- and β-globin, and those of erythroblast conventional markers, such as CD71, and CD235a, were also higher in the Fe-treated group than that in the non-Fe-treated group, suggesting the beneficial effects of Fe in development of mature erythroid lineages. In addition, markers for megakaryocyte erythrocyte progenitor (MEP) and erythrocyte progenitor (ErP) were investigated in differentiated cells by Fe treatment. CD41, vWF, and CD235a were clearly expressed in differentiated MEP cells. RUNX1 which is representative marker of definitive hematopoiesis, also clearly expressed from day 20 of differentiation. CD235a^+^ cells detached from CD41^+^ megakaryocyte were detected at day 25, CD41^+^ was disappeared, whereas Band3 with β-globin were expressed ErP at day 30 (Supplementary Fig. 3a). Genes for GATA1 and TAL1 were overall increased in non-Fe treated group and vWF was significantly decreased in Fe treated group at day 30. The genes including ANK1 and MYB were relatively increased according to differentiation of cells and Fe treatment. Markers for heme synthesis, IRP2, ALAS2, and ferrochelatase were significantly increased in Fe treated group at day 30 (Supplementary Fig. 3b).

### Single-cell RNA sequencing

**Fig. 5:**
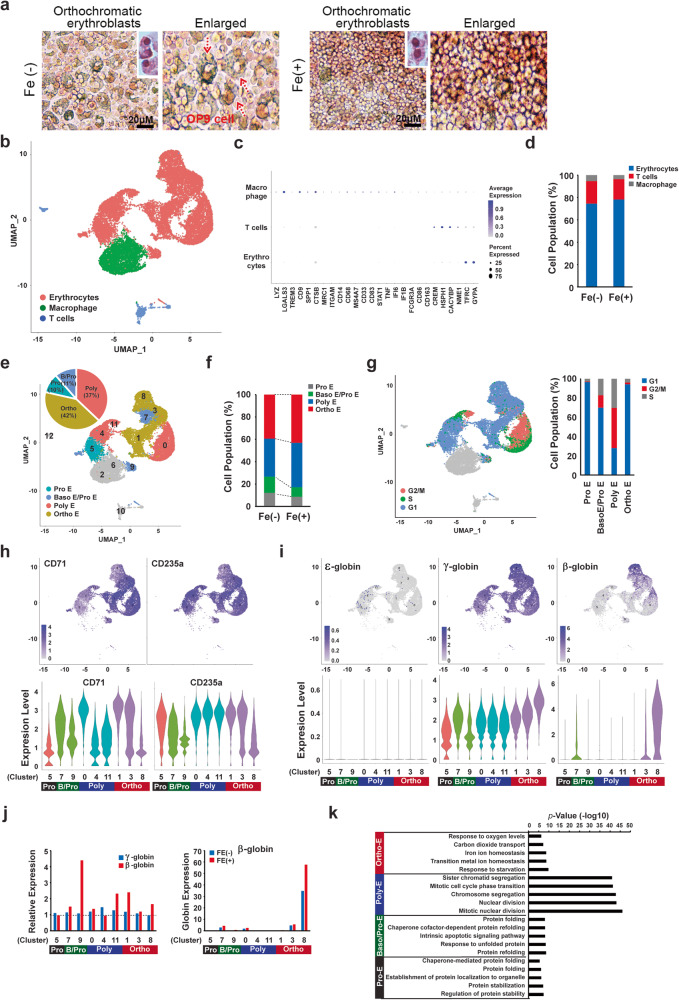
Enrichment of functional transcripts was observed in β-globin^+^ cells via single-cell RNA sequencing. **a** Erythroblasts derived after co-culturing with OP9 stromal cells were primarily characterized as orthochromatic erythroblasts before enucleation at day 33. Red arrows indicated OP9 stromal cells. Scale bar = 20 µM. Single-cell transcriptomes of differentiated erythrocytes and their distinct biomarkers observed after Fe treatment or in the absence of treatment. **b** Relative positions of the indicated cell population in the UMAP plot. **c** The dot plot of signature genes for each cell classification. **d** Stacked bar plot of proportion of the cell types. **e** The distribution of erythroblast differentiation stages on UMAP plot. Pro E, Baso E/Pro E, Poly E, and Ortho E are shown in distinct colors. The pie chart shows the percentage of cells in different cell stages. **f** Stacked bar plot of proportion of the cells in different differentiation stages on UMAP plot. **g** Cell cycling phases identified by Seurat. Stacked bar plot indicates the proportion of the cells in different cell cycle phases. **h** Gene expression heatmaps for representative erythrocyte markers (CD235a and CD71) overlaid on UMAP plots. The lower panel displays a violin plot of transcripts of CD235a and CD71 in each cluster. **i** Gene expression heatmaps for indicated globin genes overlaid on UMAP plots. The lower panel displays a violin plot of indicated genes in each cluster. **j** The relative expression of genes encoding χ- and β-globin in Fe-treated erythrocytes compared to those in the control. The right panel presents the expression level of genes encoding β-globin. **k** Enriched GO terms and *P* values of the four stages of cells.

Based on the transcript and protein levels of β-globin in erythroblasts, the molecular movement of β- globin was observed in response to Fe treatment despite the low protein levels. To further investigate whether the transcript levels of molecules associated with to RBC functional maturation, including oxygen and gas transport, are correlated to Fe treatment, single-cell RNA sequencing was performed using orthochromatic erythroblasts that maintained saturated status for hemoglobin synthesis before enucleation on day 33. As shown in Fig. 5a, orthochromatic erythroblasts in each group were observed on OP9 stromal cells. Next, we performed single-cell RNA-seq to identify the functional status of the RBCs at the transcriptional level derived under conditions with or without Fe. scRNA-seq libraries are constructed based on a rhapsody platform of 8785 cells derived from control and 10,412 cells derived from Fe-treated erythroblasts, which are obtained using Seurat-based rigorous quality control. The UMAP analysis showed that erythroblasts derived from hPSCs could be partitioned into 13 transcriptionally distinct clusters (Supplementary Fig. 4a). To verify whether Fe enhanced RBC maturation and developmental progress, cell differentiation trajectory was reconstructed using Monocle. Clusters 2 and 12 belonged to a group of cells observed at the earliest differentiation stage, while clusters 0 and 11 included cells at a very late differentiation stage (Supplementary Fig. 4b, c). However, no distinct differences in Fe-specific developmental status were observed in hPSCs-derived erythroblasts (Supplementary Fig. 4d). Gene Ontology (GO) analysis based on differentially expressed genes (DEGs) was performed to gain insights into the related biological processes in each cluster (Supplementary Fig. 4e). GO terms for each cluster displayed significantly enriched DEGs, which were connected but not limited to, chromosome segregation, ribonucleoprotein complex biogenesis, and response to endoplasmic reticulum stress. Clearance of unnecessary organelles, such as mitochondria, is required for the correct formation of mature red blood cells. These important biological processes were well represented in cluster 8, which showed enriched associated GO terms, such as autophagy of mitochondrion, mitochondrion disassembly, and organelle disassembly. Moreover, genes for oxygen transport, a function of hemoglobin, were highly enriched as well, demonstrating the development of functional RBCs from hPSCs.

Using marker-based annotation based on the expression and distribution of lineage-specific marker genes, we aimed to designate an identity of each population, in where cells with matching labels shared many lineage-specific genes, and then visualize cells on UMAP (Fig. 5b, c). The analyzed cells could be assigned into three cell types, including erythrocytes, macrophage, and cells with T-lineage characteristic, but majority of them belonged to erythroid population. In addition, we found no significant comparative differences in the distribution of cell types between the Fe-treated samples and the control (Fig. 5d). Subsequently, we subgrouped the erythroid population into differentiation stages corresponding to Pro E, Baso E, Poly E, and Ortho E using Seurat, based on the previously reported expression of marker genes during human erythroid maturation^28–30^. Our analysis revealed that 42% of erythrocytes belonged to Ortho E, 37% of cells were categorized as Poly E, and 21% of cells were classified as Baso E and Pro E cells (Fig. 5e). In addition, we observed that Ortho E and Poly E were more prevalent in the distribution of erythroid populations under the Fe-treated samples compare to the control (Fig. 5f). Furthermore, we performed cell cycle analysis based on the expression pattern of genes involved in regulating the cell cycle using Seurat and visualized on the UMAP (Fig. 5g). The cell cycle pattern exhibited high heterogeneity across different cell stages, with Poly E being more enriched in the G2/M and S phases, while Ortho E and Pro E cells were mostly in the G1 phase.

Next, we compared the maturation status based on the expression of erythroid lineage markers [TFRC (CD71)/GYPA (CD235a)]. Both CD71 and CD235a were co-expressed in most clusters, except clusters 4, 5, and 11, which were enriched with CD235a expression (Fig. 5h and Supplementary Fig. 4f). However, no comparative increase in the mRNA levels of erythroid lineage markers in proportion to each cluster was observed in Fe-treated samples compared to that in the control (Supplementary Fig. 4g). The most important biological process in the RBC maturation is globin expression, signified by the transition of ε- and γ-globin into β-globin during developmental stages between primitive and definitive hematopoiesis. However, hPSCs dominantly produced erythroids that expressed ε- and γ-globins. To validate whether Fe is involved in the regulation of globin switching, we monitored expression patterns of all globin genes. γ-globin was highly expressed in most clusters, whereas embryonic ε-globin was barely detected. Surprisingly, clusters 3 and 8, corresponding Ortho E cells, showed enriched expression of adult β-globin. This implies that erythrocytes derived using the current method showed a transition from fetal to adult globin (Fig. 5i and Supplementary Fig. 4h). We noted a marked increase in the expression of genes encoding β-globin but not γ-globin upon treatment with Fe, speculating the ability of Fe to transition into adult hematopoietic specifications (Fig. 5j and Supplementary Fig. 4h). We believe this to be the first comprehensive demonstration of hPSCs overcoming intrinsic embryonic programs and showing the preparatory stage for mature red blood cells with adult globin expression.

To gain insights into the underlying biological processes, we performed GO analysis based on the DEGs among the four groups (Ortho E, Poly E, Baso E/Pro E, and Pro E) (Fig. 5k). The GO analysis revealed that the DEGs in Baso E/Pro E and Pro E cells were significantly enriched for processes associated with protein folding and stabilization, while Poly E cells exhibited significantly enriched genes sets related to cell cycle regulation, including sister chromatid segregation, mitotic cell cycle phase transition, and chromosome segregation. Furthermore, we observed that important biological processes related to erythrocytes, such as oxygen levels, carbon dioxide transport, and iron ion homeostasis, were highly enriched in Ortho E. Notably, Ortho E cells exhibited increased β-globin expression, implicating the development of functional RBCs from hPSCs.

**Fig. 6:**
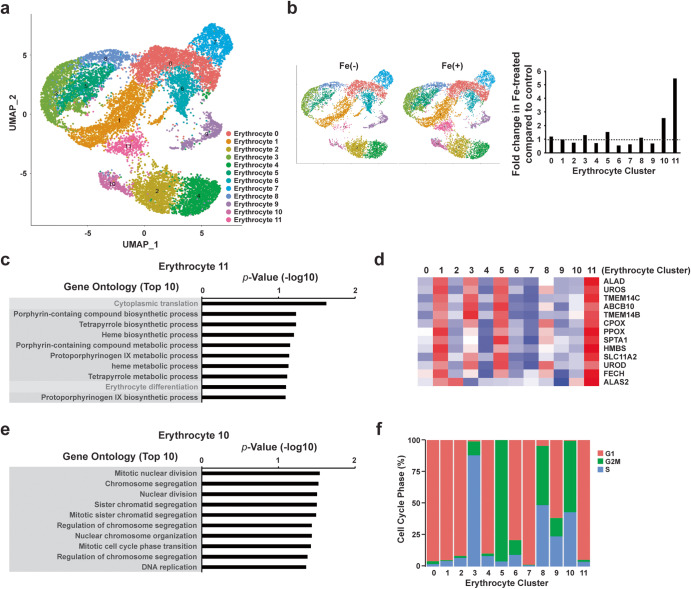
The scRNA-seq reveals a distinct population featuring the characteristics of erythrocyte maturation in the Fe-treated group. **a** UMAP visualization of heterogenous clusters of erythrocytes; 12 erythrocyte clusters were identified based on expressed UMIs. **b** Fold-change of each erythrocyte cluster in Fe treated group compared to control (right). UMAP plots between control vs Fe-treated group (left). **c** Enriched GO terms and *P* values of erythrocyte 11. **d** Heatmap displaying the mean expression of genes that represent the heme biosynthetic process within each erythrocyte cluster. **e** Enriched GO terms and *P* values of erythrocyte 10. **f** Percentage of computationally assigned cell cycle (G1, S, and G2/M) phases in each of the 12 erythrocyte clusters.

To more clearly elucidate the effect of Fe on RBC maturation, we further cataloged the heterogeneity of erythrocytes by performing unsupervised clustering of scRNA-seq data. We focused conservatively on the erythroblasts and identified 12 distinct erythrocyte clusters based on transcriptional profiles (Fig. 6a). Comparing the proportion of erythrocyte clusters between the control and Fe-treated groups revealed that certain erythrocyte clusters, specifically 10, and 11, were significantly enriched in the Fe-treated group (Fig. 6b). Notably, erythrocyte 11 exhibited a significant increase in Fe-treated groups and was uniquely enriched in genes associated with heme biosynthetic process, which is closely related to hemoglobin synthesis, and erythrocyte differentiation (Fig. 6c, d), implicating contribution of Fe in the maturation of erythrocytes by regulating globin synthesis. Erythrocyte 10, which showed a second highest enriched proportion compared to control, was subjected to gene ontology analysis, revealing several gene sets relevant to cell cycle regulation (Fig. 6e). The analysis of genes related to cell cycle phases showed that this erythrocyte 10 was particularly distinct from the other erythrocyte clusters due to its enrichment in G2/M and S cells (Fig. 6f). In addition, we observed an increase in G2/M or S phase cells in erythrocyte 3, 5, and 8. Notably, the proportion of cell population in these clusters was higher in the Fe-treated groups compared to control group. These results suggest that the significantly increased cumulative number of erythroblasts in Fe-treated groups (Fig. 2b) is mediated by transcriptional regulation resulting from the addition of Fe. Taken together, these results highlight that the transcriptional programs induced by Fe treatment contribute to the maturation of erythrocytes by regulating globin expression and increasing the number of differentiated erythroblasts derived from hPSCs through cell cycle control.

### In vivo maturation of erythroblasts

**Fig. 7:**
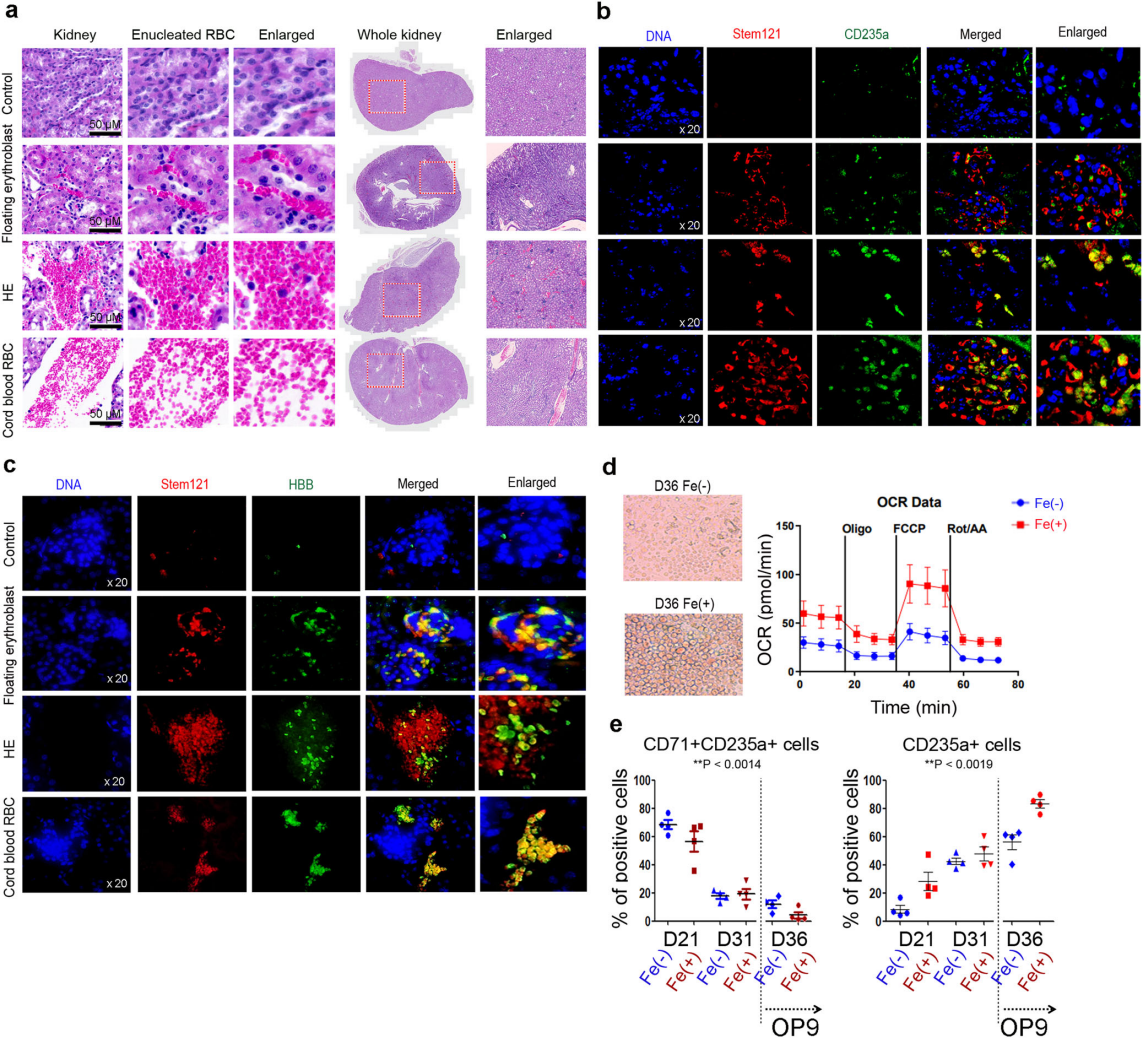
Erythroblasts were differentiated into biconcave disk-shaped RBCs with β-globin expression in vivo. **a** Immunostaining of erythroblasts, CB-RBC, and HE showed mature RBCs in the kidneys of NSG mice. The mice were euthanized on day three after cell injection, and kidney tissues were stained with H&E. Scale bar = 50 µM **b** Immunohistochemistry data showed that human-specific markers stem121 and CD235a were co-expressed without DAPI in the kidney tissues. Magnification ×20. **c** Immunohistochemistry data also showed that human-specific markers stem121 and β-globin were co-expressed without DAPI in kidney tissues. Data clearly showed biconcave disk-shaped RBCs, implying the development of mature RBCs. Magnification ×20. **d** The capacity of mitochondrial respiration was increased in the Fe-treated group than that in the non-Fe-treated group. *n* = 9 per group. **e** Flow cytometry data of CD71^+^CD235a^+^ cells and CD235a^+^ erythroid cells in differentiated erythroblasts and erythrocytes on OP9 stromal cells. The percentage of CD71^+^CD235a^+^ cells decreased rapidly and that of CD235a^+^ erythroid cells increased upon co-culturing with OP9 stromal cells. Overall, the Fe-treated group displayed a higher increase frequency during differentiation, suggesting the development of an optimized culture condition supplemented with ferric citrate for erythroid lineages. Data are represented as means ± SEM derived from two independent experiments performed in duplicate. *n* = 4 per group.

We next examined the capacity of erythroblasts and HE to differentiate into mature erythroid cells in vivo. Differentiated HE and erythroblasts were injected into the kidney, skin, and muscles of NSG mice. The mice were euthanized three days after injection. Unexpectedly, the group injected with HE cells showed that injected HE cells were engrafted in the kidney and robustly produced mature RBCs with a biconcave disk shape. Differentiated erythroblasts and CB-derived RBCs were also observed in the kidney. Mature RBCs were observed in both groups (Fig. 7a). Similar to in vivo maturation of erythroblasts in the kidney, injected erythroblasts and HE cells were engrafted into the skin and muscles, and erythroblasts developed into mature RBCs (Supplementary Fig. 5). However, the number of cells engrafted in the kidney was the highest compared to that of other tissues due to supplementation with abundant EPO and microenvironmental cytokines. To confirm if these RBCs originated from human PSCs, immunohistochemistry analysis was used to evaluate human-specific markers Stem 121 and CD235a in kidney tissues. Stem121^+^CD235a^+^ RBCs were clearly co-expressed in the kidney tissues. These cells were detected without a nucleus, implying the development of enucleated RBCs (Fig. 7b). Stem121^+^CD235a^+^ biconcave disk-shaped RBCs were also detected in muscle tissues (Supplementary Fig. 6). To test if enucleated RBCs were matured under in vivo conditions, we examined the expression of human-specific β-globin in kidney tissues. β-globin, which requires a long time to mature, was clearly expressed in tissues. Notably, suspended erythroblasts and CB-RBCs showed that stem121^+^β-globin^+^ markers were almost co-expressed in the kidney, whereas not all HE-derived RBCs expressed β-globin, and these erythrocytes were still developing into immature erythrocytes (Fig. 7c). To verify that erythroblasts, which can rapidly acquire β-globin in vivo, were cultured under optimized conditions involving Fe treatment for hemoglobin synthesis and switching, we performed oxygen consumption rate (OCR) analysis. The capability of mitochondrial respiration was significantly higher in the Fe-treated group than that in the non-Fe-treated group. These results show the beneficial effects of Fe on the development of RBCs from PSCs based on morphological and maximal respiration observed with upregulation of relevant transcripts (Fig. 7d). Consistent with OCR results, the number of CD71^+^CD235a^+^ cells in mature erythroid CD45^-^ fraction was decreased significantly based on the treatment day, and the number of cells was remarkably decreased in the OP9 stromal cell co-culture system with Fe treatment. Meanwhile, enriched CD235a^+^ cells in CD45^-^ erythroid cells increased remarkably in the OP9 stromal cell co-culture system with Fe treatment (Fig. 7e).

Taken together, we showed the beneficial effects of ferric citrate on the development of erythroblasts into mature RBCs with β-globin transcriptional expression. The culture protocol for pure CD34^+^ HE sorting was robust and reproducible for the production of erythroblasts. The present study might be helpful for investigating mechanisms underlying erythropoiesis and hematological diseases in regenerative medicine. Furthermore, a simple and feasible approach for manufacturing RBCs under in vitro conditions may be developed for clinical use.

## Discussion

Hematopoiesis, especially erythropoiesis, is a complex process involving balanced interactions among cytokines, matrix factors, and enucleation mediated by pivotal transcription factors. Owing to the inaccessibility of human embryonic cell sources, the cellular and molecular events driving erythropoiesis during human early development remain largely unknown. The development of proper ex vivo systems to model the cellular steps of erythropoiesis in human PSCs is challenging owing to insufficient data on the human fetus. Despite these difficulties, we used a human embryonic stem cell line and designed a simple and reproducible culture system using ferric citrate treatment. For the initiation of erythropoietic development, CD34^+^CXCR4^-^CD73^-^ definitive HE cells were first established by CD34^+^ cell sorting (supplementary Fig. 1a), and the growth factors EPO, FLT3L, and SCF, which are crucial for human definitive erythropoiesis, were added under ex vivo conditions^31^. Owing to the continuous maturation of erythroblasts from HE over four weeks, 1.858 × 10^9^ erythroblasts could be harvested from 1.4 × 10^6^ HE cells, which corresponds to the production of 4645 RBCs from one ES cell via the addition of Fe (Fig. 2b). Previous studies have identified some requirements for RBC differentiation from PSCs in culture systems. The primary requirements include a cost-effective system obtained by the addition of cytokines, a feeder- and serum-free system for clinical applications, a large-scale proliferation of cells, high efficiency for enucleation, and functional maturation via induction of β-globin expression^32–36^. Although many trials have been conducted, the guarantee of an RBC production method that can enter clinical trials remains unclear.

Transferrin recycling undergoes pH changes. Holo-transferrin be bound to its receptor on the cell surface, then rapidly internalized into endosomal vesicles and ultimately was released the iron from the transferrin under low pH 5.5^37^. Apo-transferrin released from the receptor can be recycled to bind additional iron to deliver more iron into the cells. Both holo-transferrin formation and transferrin endocytosis require a base pH of 7.2–8.5. Since pluripotent stem cell-derived erythroid lineage cells massively increase in numbers, proper differentiation and growth failed unless sufficient iron was supplied for proliferation. The uptake of holo-transferrin in Apel II medium, which maintains pH 7.2 (as mentioned in the product certificate of analysis from stem cell technologies), is better for the endocytosis of erythroblasts^38^. We showed that pH condition of Apel II media leads to loading iron onto apo-transferrin and transferrin uptake. In addition, we expected that CD71 expressing erythroblasts may need apo-transferrin, which has a strong capacity to bind substances, such as iron and metal ions, thereby resulting in the increase in iron uptake by erythroblast. Similar to previous data^33,39^, cumulated erythroblasts increased around 200-fold according to the serial concentration of holo-transferrin (supplementary Fig. 7). It suggests that a higher proliferation effect can be expected if apo-transferrin and ferric citrate separately. We aimed to optimize iron and transferrin formulations for industrial production. To end this, recombinant transferrin free of fibrinogen was used in present study, resulting in RBC with cost-effective methods.

Erythroblasts can uptake iron from transferrin. Transferrin commonly exists in three forms: holo-transferrin, apo-transferrin, and partial iron-loaded transferrin. Among these, holo-transferrin has been commonly used to generate RBCs from PSCs. However, we used the ferric iron and transferrin separately, thereby enhancing the possibility of cost-effectiveness in large-scale production. We also demonstrate that different formulations of iron complexes and apo-transferrin can support the differentiation of erythroblasts in culture, which suggests optimizing iron and transferrin formulation along with using cost-effective methods for industrial production.

In particular, the expression of β-globin in RBCs is crucial for protecting the cell membrane, which is prone to hemolysis. Although most erythrocytes successfully complete enucleation, β-globin is rarely expressed. For enucleation in erythrocytes, the support of BM microenvironmental factors, non-hematopoietic cells, or contact of macrophages in erythroblastic island/erythroblast macrophage protein might be required by cellular interactions^4,39,40^. OP9 stromal feeder cells that acted as a specific niche produced fully matured erythroblasts, particularly with enucleation potential. However, our culture system was not capable of inducing high expression of β-globin, like adult RBCs. Ferric citrate played a central role from the early HE developmental stage to prime definitive HE development for producing erythroblasts containing the transcript factors for β-globin, as observed by a single-cell RNA sequencing assay. This enrichment in the expression of genes related to oxygen and gas transport and iron homeostasis showed that β-globin expression was substantially higher in Fe-treated erythroblasts than that in non-Fe-treated groups, suggesting the crucial role of ferric citrate in RBC maturation, especially in mediating the switch to β-globin expression from γ-globin. The fetal globin was expressed in early definitive erythrocytes in the fetal liver with a low expression of adult β-globin^41^. PSC-derived erythroblasts showed a high expression of embryonic (epsilon) and fetal (gamma, γ) globin, with little adult β-globin expression^3^. To date, no reports exist on whether Fe supplementation is relevant for improving the expression of β-globin in erythroblasts without feeder cells. Although we did not observe a significant increase in β-globin protein expression with Fe treatment, single-cell RNA sequencing data showed an increase in β-globin transcript level. In our differentiation system, single-cell RNA sequencing data demonstrated an increase in β-globin transcript levels with the addition of Fe. The development of RBCs with β-globin relies on pivotal iron regulators, Iron Regulatory Proteins (IRP) 1 and 2, in heme synthesis. IRP2 deficiency results in decreased transferrin receptor levels, leading to insufficient heme biosynthesis in erythroblasts^42,43^. In culture system, both IRP2 and erythroid 5-aminolevulinate synthase (ALAS2), the first enzyme in heme synthesis, were highly upregulated in differentiated cells (Supplementary Fig. 3). During in vitro maturation of cultured RBCs, the generation of RBCs from BM-HSCs typically takes ~21 days and can be accelerated by TGF-β1 treatment^44^. The life span of erythrocytes produced in postnatal BM is ~120 days, and erythrocyte precursors take ~7 days to mature^45,46^. In the present study, we demonstrated the rapid development of biconcave disk-shaped RBCs with human-specific markers and β-globin in vivo. Mature RBCs were observed within three days in vivo, implying the role of micro-environmental factors in the development of RBCs. We successfully developed the erythrocyte with β-globin transcript level, but functional assay still remains to be investigated using completion of protein level. Next, we will apply the 3-dimentional architecture system mimicking BM which has abundant macrophages involving erythroid maturation or spheroid vitro culture system with specified cytokines^47^.

Taken together, erythroblasts showed a high level of β-globin transcripts when cultured with Fe and develop into mature RBCs immediately after injection in vivo. We will further examine the factors that can regulate maturation of erythroid lineage cells using erythroblasts treated by Fe in an advanced ex vivo culture system for maintenance of cell membrane integrity after enucleation. Finally, we aim to establish a stable protocol for the development of clinically applicable RBCs.

## Methods

### Ethics statement

All experiments were performed with authorization from the Institutional Review Board for Human Research at the CHA University (1044308-202204-LR-023-02). All animal protocols were reviewed and approved by the Institutional Animal Care and Use Committees of the CHA University (IACUC220090), and all animal procedures were performed in accordance with approved guidelines and regulations.

### Culture and differentiation of human PSCs to HE

Human PSCs (CHA52 embryonic stem cell line, PSCs) were seeded onto Matrigel-coated plates with mTeSR™1 (85850, Stem Cell Tech) for differentiation into HE. To induce the development of mesoderm, the cells were treated with Apel 2 medium (5275, Stem Cell Tech) supplemented with 3 nM CHIR-99021 (S2924, Selleck Chemicals), 20 ng/ml VEGF_165_ (100-20, Peprotech), and 25 ng/ml BMP-4 (HZ-1045, HumanKine) for three days^48^. To induce the formation of HE, cells were treated with Apel 2 medium supplemented with 250 ng/ml SCF (300-07, Peprotech), 20 ng/ml VEGF_165_ (100-20, Peprotech), 25 ng/ml BMP-4 (HZ-1045, HumanKine), 200 ng/ml Flt3-Ligand (300-19, Peprotech), 100 ng/ml TPO (300-18, Peprotech), and 20 ng/ml EPO (100-64, Peprotech) as described previous papers^49–55^. The differentiated cells were regularly observed under a microscope (IX73, OLYMPUS, Japan).

### Purification and culture of CD34^+^ cells

Isolation of CD34^+^ cells from PSC-derived HE was performed by magnetic-activated cell sorting (MACS) using the CD34 MicroBead Kit (130-046-703, Miltenyi Biotec) according to the manufacturer’s instructions. Sorted CD34^+^ cells were cultured in the Apel 2 medium containing several cytokines as shown in Fig. 1a. Among these, 400 µg/ml of ferric ion (F3388, Merck), 50 ng/ml of IGF-1 (AF-100-11, Peprotech), 50 ng/ml of folic acid (F8758, Merck), and 50 ng/ml of transferrin (10652202001, Roche) of our 4-stage erythroid culture system were added till 21 days. Ferric citrate and transferrin were primarily added for the differentiation of PSCs into erythroblasts. On day 21, cells were transferred to a mature medium without EPO, TPO, and IGF-1. The medium was replaced with a fresh medium 2–3 times per week. To promote erythroid differentiation, proliferating erythroblasts were transferred on the OP9 stromal cells, and the maturation of the cells was observed along with β-globin expression.

### Antibodies and flow cytometry

To analyze erythroblasts, the harvested cells were resuspended in 100 µl of 0.5% FBS in PBS and stained with antibodies. After washing with rinsing buffer, the cells were analyzed using a BD Accuri C6 plus (BD Biosciences), and data were evaluated using the BD Accuri C6 plus software. The following antibodies were used for this study: APC-conjugated mouse anti-human CD45 (555485, BD Pharmingen™), PE-Cy™7-conjugated mouse anti-human CD235a (563666, BD Pharmingen™), FITC-conjugated mouse anti-human CD71 (555536, BD Pharmingen™), rabbit anti-human fetal globin (ab283313, Abcam), PE-conjugated rabbit IgG (A11012, Invitrogen) (used as a secondary antibody), and FITC-conjugated mouse anti-human β-globin (sc-21757, Santa Cruz).

### Immunocytochemistry (ICC)

Immunocytochemistry (ICC) was conducted as previously described^56^. Cells were initially fixed with 2% PFA and permeabilized with 0.25% Triton X-100 for 20 min at room temperature. After washing with PBS, the cells were blocked with 5% serum and were subjected to staining with primary antibodies, followed by incubation with secondary antibodies. For erythroblast marker analysis, CD235a (ab129024, Abcam) and CD71 (ab9179, Abcam) were used as primary antibodies. For staining of hemoglobin, anti-human fetal globin (ab283313, Abcam) and anti-β-globin (HPA043234, Sigma), Runx1 (Abcam, ab35962), vWF (Abcam, ab11713), CD41 (Santa Cruz, sc-21783) and Band3 (Proteintech, 28131-1-AP) were used as primary antibodies. A secondary antibody was used to detect primary signals. DAPI was used for nuclear staining, and the cells were visualized under a fluorescent microscope (IX73, OLYMPUS or Axio Imager.a2, Carl Zeiss or Zeiss LSM 880 confocal laser scanning microscope with Airyscan and LSM 880 Image software; CLSM, Carl Zeiss, Germany).

### Immunohistochemistry

Immunofluorescence analysis was performed using paraffin sections with a thickness of 8 μm. After deparaffinization and rehydration, permeabilization was performed with PBS containing 0.5% Triton X-100 for 15 min. The sections were blocked with 2% BSA in PBS for 60 min at room temperature and incubated with primary antibodies diluted in PBS containing 2% BSA and 1% Tween 20 at 4 °C overnight. The following primary antibodies were used in this study: 1:100 CD235a (Ab129024, Abcam), 1:100 STEM121 (Y40410, Takara), and human β-globin (HPA043234, Merck). The next day, after washing three times with 1% Tween 20 in PBS, the samples were incubated with secondary antibodies for 60 min at room temperature in the dark. The following secondary antibodies were used in this study: 1:400 anti-rabbit IgG Alexa Fluor 488 (A11008, Invitrogen) and 1:400 anti-mouse IgG Alexa Fluor 555 (A21422, Invitrogen). After washing again with 1% Tween 20 in PBS, the sections were stained with 1:5000 DAPI (D9542, Millipore) in PBS for nuclear staining and then mounted on slides. Imaging of the samples was performed using a Zeiss LSM 880 confocal laser scanning microscope with Airyscan and LSM 880 Image software (CLSM, Carl Zeiss, Germany).

### Colony-forming unit (CFU) assay

To evaluate whether the PSC-derived HE demonstrated the function or potency of hematopoietic progenitors, 2 × 10^4^ PSC-derived HE cells were suspended in 500 µl of MethoCult™ (H4434, Stem Cell Technologies) and cultured for 14 days. The formation of colonies and tube-like structures was monitored under a microscope (IX73, OLYMPUS).

### Cytospin preparation and Wright-Giemsa staining

To stain the erythroid lineage cells from PSC-derived HE, the 10^5^ cells in 300 mL were used for cytospin preparations on coated slides using a cytological centrifuge (B18052134, Cytospin-TXT3). The slides were stained with Wright-Giemsa Solution (ab245888, Abcam) according to the manufacturer’s protocol. Briefly, the slide was fixed with methanol for five minutes and then flooded with Wright-Giemsa Solution for five minutes. After washing with distilled water, the slides were rinsed with PBS and covered using a mounting solution. The cells were imaged using a microscope (Axio Imager.a2, Carl Zeiss).

### Quantitative RT-PCR (qRT-PCR)

Total RNA was extracted from harvested cells using TRIzol reagent (Ambion) according to the manufacturer’s instructions. cDNA was synthesized using a reverse transcriptase kit (RT200, Enzynomics). qRT-PCR was performed with SYBR Green (RT500M, Enzynomics) using a Real-Time System (Bio-Rad, CFX96™). Relative mRNA expression of target genes was calculated using the comparative CT method. All target genes were normalized to *18s* in multiplexed reactions performed in triplicate. Differences in CT values were calculated for each target mRNA by subtracting the mean value of *18s* (relative expression = 2^−ΔCT^). Information on primers used in this study is provided in Table 1.

**Table 1:** Primers and probes for quantitative RT-PCR.

Genes		Primers and probes (5’–3’)
human	Forward	GAAACGGCTACCACATCCAAG
*18s*	Reverse	CGGGTCGGGAGTGGGT
human *embryonic globin*	Forward	TACTGCTGAGGAGAAGGCTG
	Reverse	CAATGGCGACAGCAGACAC
human *alpha globin*	Forward	ATGTTCCTGTCCTTCCCCAC
	Reverse	GCTCACAGAAGCCAGGAACT
human *beta globin*	Forward	ACATTTGCTTCTGACACAAC
	Reverse	ACAGATCCCCAAAGGAC
human *CD71*	Forward	ATCGGTTGGTGCCACTGAATGG
	Reverse	ACAACAGTGGGCTGGCAGAAAC
human *CD235a*	Forward	ATATGCAGCCACTCCTAGAGCTC
	Reverse	CTGGTTCAGAGAAATGATGGGCA
human *GATA1*	Forward	ATCACACTGAGCTTGCCACA
	Reverse	CAGGCCAGGGAACTCCA
human *TAL1*	Forward	ACCACCAACAATCGAGTGAAGAGGAGAC
	Reverse	CTGTTGGTGAAGATACGCCGCACAA
human *MYB*	Forward	CAGGAAGGTTATCTGCAGGAGTCTTCAAAA
	Reverse	CTATAGGCGGAGCCTGAGCAAAA
human *IRP1*	Forward	GAAAGTCCAGGTCAAGCTGGATAC
	Reverse	CTTGGCCATCTTGCGGATCATGTAG
human *IRP2*	Forward	CCTTGGGCCTCTCCGGTAGAGAAAC
	Reverse	CAAACGAAGCAATCACGCTGAATAC
human *ANK1*	Forward	TCAGGGGGTCCCTGTGCTTTGT
	Reverse	GCCCTGCTCATCCGTGAATTGC
human *ferrochelatase*	Forward	GATGAATTGTCCCCCAACAC
	Reverse	GCTTCCGTCCCACTTGATTA
human *ALAS2*	Forward	GGAGCGTGATGGAGTTATGC
	Reverse	GATTCTAAAGCCCCAGAGAGC
human *vWF*	Forward	CCTTGGTCACATCTTCACATCAC
	Reverse	TCATTGGCTCCGTTCTCATCAC

### Multiplexing individual samples for scRNA-seq

To multiplex samples, each sample was tagged with antibody-polyadenylated DNA barcode for human cells (BD Biosciences, cat no. 633781). Briefly, cells were stained with multiplexing antibody for 20 min at room temperature, and then washed 3 times by using stain buffer (BD Biosciences, cat no. 554656). After final washing, samples were gently resuspended in cold sample buffer (BD Biosciences, cat no. 633731), counted with a LUNA-FX7™ Automated Fluorescence Cell Counter (logos biosystems), and then pooled.

### Single-cell RNA sequencing library construction

Single-cell capture was performed using BD Rhapsody Express instrument according to the manufacturer’s instrument (BD Bioscience). Briefly, 15,000 pooled cells from each sample in cold sample buffer were loaded into the BD Rhapsody cartridge (BD Biosciences, cat no. 633731). After cells separation, cell barcode-magnetic beads were loaded into the cartridge. Cells were lysed and the mRNA capture bead was retrieved. cDNA synthesis and Exonuclease I treatment were performed on the mRNA capture beads using BD Rhapsody cDNA kit (BD, cat no. 633773).

### Library preparation for single-cell RNA sequencing

According to the ‘mRNA Whole Transcriptome Analysis and Sample Tag Library Preparation’ protocol (BD Bioscience), scRNA-seq libraries were constructed using BD Rhapsody WTA amplification kit (BD, cat no. 633801). Briefly, for whole transcriptome analysis (WTA) library, cDNA was sequentially subjected to the random priming and extension (RPE), RPE amplification, and index PCR. And for sample tag library, cDNA was sequentially subjected to the nested PCR (PCR 1 and PCR 2), and index PCR.

### Single-cell RNA sequencing and BD Rhapsody

The purified WTA and Sample tag libraries were quantified using qPCR according to the qPCR Quantification Protocol Guide (KAPA), and qualified using the Agilent Technologies Bioanalyzer DNA high Sensitivity chip (Agilent Technologies). And then libraries were pooled and sequenced using HiSeq platform (Illumina), and 150 bp paired-end reads were generated. The sequencing depth of WTA library was about 10,000 reads/cell and sample tag library was about 120 reads/cell. Raw sequencing data (FASTQ) were processed through BD Rhapsody WTA Analysis Pipeline v1.10.1 using human reference genome (GRCh38).

### Data processing

Downstream analysis was carried out with R v4.0.3 and Seurat v3.2.2^57^. Quality control, cell filtering, normalization, and clustering were carried out based on Seurat. We used SCT as the normalization method and performed PCA on the preprocessed matrix with first 16 principal components for clustering. SNN algorithm was used to perform clustering with a resolution of 0.4 and visualization is performed using Uniform Manifold Approximation and Projection (UMAP). Pseudotime trajectory analysis was performed using R package Monocle2^58,59^, used to construct the branched pseudotime trajectory (Monocle2 documentation). Gene ontology (GO) and Kyoto Encyclopedia of Genes and Genomes (KEGG) were performed with cluster Profiler v3.18.1 in R v4.0.3^59^, which supports statistical analysis and visualization of functional profiles for genes and gene clusters. Cell cycle stages were predicted using the CellCycle Scoring function based on G2/M and S phase marker expression^60^. The proportion of cells assigned to each phase within each cluster was then determined.

### In vivo studies

NOD/ShiLtSz-scid/IL2Rg^null^ (NOD.Cg-*Prkdc*^scid^Il2rg^tm1Wjl^/SzJ or Nod *scid* gamma (NSG)) mice were purchased from the Jackson Laboratory and housed in ventilated micro-isolator cages in a high-barrier facility under specific pathogen-free conditions. The animals were provided with ad libitum access to autoclaved water and irradiated food. To examine the engraftment of erythroblasts, CB-RBCs, and HE, 5 × 10^5^ cells were injected into the kidney, ear skin, and muscles. After three days, tissues were obtained from euthanized mice and subjected to hematoxylin and eosin staining and immunohistochemistry analysis of β-globin and CD235a.

### Mitochondrial oxygen consumption rate

The mitochondrial oxygen consumption rate (OCR) was measured using a Seahorse XF-96 analyzer (Agilent Technologies, Santa Clara, CA, USA) in 96-well plates. On the day before the OCR measurement, the sensor cartridge was placed in an incubator at 37 °C without CO_2_ for 24 h. The cells were resuspended in serum-free XF assay medium (Agilent Technologies) with a pH of 7.4 supplemented with 10 mM glucose, 2 mM glutamine, and 1 mM pyruvate. CD71^+^CD235a^+^ cells pre-treated with Fe or those without pretreatment were seeded at a density of 3 × 10^5^ onto poly-L-lysine (Sigma-Aldrich, St Louis, MO, USA)-coated wells to enable cell attachment and assayed in an XF assay medium. Mitochondrial OCR (pmoles/min) was measured using real-time injections. For mitochondrial OCR, the cells were placed in the XF assay medium and injected with oligomycin (1 µM), carbonyl cyanide-4-(trifluoromethoxy) phenylhydrazone (2 µM), and rotenone/antimycin (0.5 µM). All chemicals were purchased from Agilent Technologies. Calculations for individual metabolic parameters were performed as described previously^60^.

### Statistical analysis

All results were presented as the mean ± SEM. Statistical analyses were performed using the Mann–Whitney U test for comparisons between two groups and the Kruskal–Wallis ANOVA test for >2 groups. Values of *P* < 0.05 were considered statistically significant. The GraphPad Prism ver. 4 software (GraphPad Software, La Jolla, CA, USA) was used for statistical analysis.

### Reporting summary

Further information on research design is available in the Nature Research Reporting Summary linked to this article.

### Supplementary information

Reporting summary

Supplementary data

## Data Availability

The data that support the findings of this study are available from the corresponding author upon reasonable request. The scRNA-seq data in this study have been deposited at the NCBI Gene Expression Omnibus under the accession number GEO: GSE227952.

## Code availability

The single cell data analysis were performed with R v4.0.3 and Seurat v3.2.2. Images were analyzed using a Zeiss LSM 880 confocal laser scanning microscope with Airyscan, microscope (IX73, OLYMPUS, Japan) and LSM 880 Image software (CLSM, Carl Zeiss, Germany). GraphPad Prism version 4 was used for statistical analysis. The software version and websites were described in the “Methods” section and all codes are available upon reasonable request.
