# Supplementary material for: Ferric citrate and apo-transferrin enable erythroblast maturation with β-globin from hemogenic endothelium
Source: NPJ Regen Med. 2023 Aug 25;8:46. doi: 10.1038/s41536-023-00320-4 (PMC10457393; doi:10.1038/s41536-023-00320-4)
Supplement: Supplementary file 1 — Reporting summary [file 41536_2023_320_MOESM1_ESM.pdf]

nature research

Corresponding author(s): Ji Yoon Lee AND Jong-Hee Lee

Last updated by author(s): July.29.2023

Reporting Summary

Nature Research wishes to improve the reproducibility of the work that we publish. This form provides structure for consistency and transparency in reporting. For further information on Nature Research policies, see Authors & Referees and the Editorial Policy Checklist.

Statistics

For all statistical analyses, confirm that the following items are present in the figure legend, table legend, main text, or Methods section.

n/a	Confirmed	
☐	☒	The exact sample size (*n*) for each experimental group/condition, given as a discrete number and unit of measurement
☐	☒	A statement on whether measurements were taken from distinct samples or whether the same sample was measured repeatedly
☐	☒	The statistical test(s) used AND whether they are one- or two-sided<br>*Only common tests should be described solely by name; describe more complex techniques in the Methods section.*
☒	☐	A description of all covariates tested
☐	☒	A description of any assumptions or corrections, such as tests of normality and adjustment for multiple comparisons
☐	☒	A full description of the statistical parameters including central tendency (e.g. means) or other basic estimates (e.g. regression coefficient) AND variation (e.g. standard deviation) or associated estimates of uncertainty (e.g. confidence intervals)
☐	☒	For null hypothesis testing, the test statistic (e.g. *F*, *t*, *r*) with confidence intervals, effect sizes, degrees of freedom and *P* value noted<br>*Give P values as exact values whenever suitable.*
☒	☐	For Bayesian analysis, information on the choice of priors and Markov chain Monte Carlo settings
☒	☐	For hierarchical and complex designs, identification of the appropriate level for tests and full reporting of outcomes
☒	☐	Estimates of effect sizes (e.g. Cohen's *d*, Pearson's *r*), indicating how they were calculated

Our web collection on statistics for biologists contains articles on many of the points above.

Software and code

Policy information about availability of computer code

Data collection	Collection of flow cytometry data: CellQuest software or Accuri C6 software. No software was used for data collection for RNA sequencing.
Data analysis	Flow cytometry : FlowJo (version 10.07) and Accuri C6 Plus flow cytometer equipped with the Cell Quest software (BD)<br>Statistical analysis and data visualization : GraphPad Prism (version 4.0,USA)<br>Fluorescence imaging : Carl Zeiss Axiovert 200M inverted microscope (Carl Zeiss) and confocal microscope (Carl Zeiss LSM 880, Germany, and IX73, OLYMPUS, Japan).<br>PCR: Real-Time System (Bio-Rad, CFX96™).<br>Single cell RNA sequencing : The BD Rhapsody WTA Analysis Pipeline Version 1.10.1 was used to map reads to the genome and transcriptome using the STAR aligner (version 2.5.2b), demultiplex cell index. Data analysis was performed in Rstudio(v1.4.1717) and R(4.0.3) with the following key libraries: writexl v1.4.1, Seurat v3.2.2, grDevices v4.0.3, dplyr v1.0.2, cowplot v1.1.0, ggplot2 3.3.2, gridExtra v2.3, stringr v1.5.0, lavaan v0.6-12, Hmisc 4.7-0, PupillometryR v0.0.4, grid v4.0.3, colorRamps v2.3.1, org.Hs.eg.db 3.12.0.<br>GO and KEGG analysis was performed in R v4.2.2 with the following libraries: clusterProfiler v4.6.0, org.Hs.eg.db v3.16.0, dplyr 1.0.10, ggplot2 v3.4.0. And gplots v3.1.3, pheatmap v1.0.12, RColorBrewer v1.1-3 were used for visualization.<br>Mitochondrial oxygen consumption: Seahorse XF-96 analyzer (Agilent Technologies, Santa Clara, CA, USA)

For manuscripts utilizing custom algorithms or software that are central to the research but not yet described in published literature, software must be made available to editors/reviewers. We strongly encourage code deposition in a community repository (e.g. GitHub). See the Nature Research guidelines for submitting code & software for further information.

Data

Policy information about availability of data

All manuscripts must include a data availability statement. This statement should provide the following information, where applicable:

- Accession codes, unique identifiers, or web links for publicly available datasets
- A list of figures that have associated raw data
- A description of any restrictions on data availability

The scRNA-seq data in this study have been deposited at the Gene Expression Omnibus under the accession number GEO: GSE227952.
All raw data used for generating figures has been deposited in the Source Data file. All other data that support the findings of this study are available from the corresponding author upon reasonable request.

Field-specific reporting

Please select the one below that is the best fit for your research. If you are not sure, read the appropriate sections before making your selection.

☒ Life sciences ☐ Behavioural & social sciences ☐ Ecological, evolutionary & environmental sciences

For a reference copy of the document with all sections, see nature.com/documents/nr-reporting-summary-flat.pdf

Life sciences study design

All studies must disclose on these points even when the disclosure is negative.

Sample size	No statistical analysis methods were used to predetermine sample size estimates. Sample size was determined to be adequate based on the magnitude and consistency of measurable differences between groups.
Data exclusions	On the few occasions where samples were excluded it was when there was clear failure of flow cytometry antibodies/ reagents
Replication	Each experiment via animals and vitro condition was repeated at least three times as described in Figure legends and manuscripts.
Randomization	NSG mice were assigned according to their genotype with IL-2R null. Litter mates after confirmation of gene knockout were used whenever possible.
Blinding	Investigators were blinded to compared group until completion of initial experiments including all.

Reporting for specific materials, systems and methods

We require information from authors about some types of materials, experimental systems and methods used in many studies. Here, indicate whether each material, system or method listed is relevant to your study. If you are not sure if a list item applies to your research, read the appropriate section before selecting a response.

Materials & experimental systems

n/a	Involved in the study	
☐	☒	Antibodies
☐	☒	Eukaryotic cell lines
☒	☐	Palaeontology
☐	☒	Animals and other organisms
☒	☐	Human research participants
☒	☐	Clinical data

Methods

n/a	Involved in the study	
☒	☐	ChIP-seq
☐	☒	Flow cytometry
☒	☐	MRI-based neuroimaging

Antibodies

Antibodies used	To address protein expression by FACS, APC-conjugated mouse anti-human CD45 (555485, BD Pharmingen™), PE-Cy™7-conjugated mouse anti-human CD235a (563666, BD Pharmingen™), FITC-conjugated mouse anti-human CD71 (555536, BD Pharmingen™), rabbit anti-human fetal globin (ab283313, Abcam), PE-conjugated rabbit IgG (A11012, Invitrogen) (used as a secondary antibody), and FITC-conjugated mouse anti-human β-globin (sc-21757, Santa Cruz). For immunostaining, CD235a (ab129024, Abcam, 1:100) and CD71 (ab9179, Abcam, 1:200), Stem121 (Y40410, Takra, 1:100) were used as primary antibodies. For staining of hemoglobin, anti-human fetal globin (ab283313, Abcam, 1:200) and anti- β-globin (HPA043234, Sigma, 1:100), Runx1 (Abcam, ab35962, 1:200), vWF(Abcam, ab11713, 1:200), CD41(Santa Cruz, sc-21783, 1:200) and Band3 (Proteintech, 28131-1-AP, 1:200) were used as primary antibodies. A secondary antibody was used to detect primary signals.
Validation	All antibodies were validated for the application and species used in this study by their manufacturers. Bead controls used for

flow.

Eukaryotic cell lines

Policy information about cell lines

Cell line source(s)	CHA-hES15 embryonic stem cell line, PSCs
Authentication	The used ES cell line was authenticated by previous paper
Mycoplasma contamination	All cell lines were not detected for mycoplasma contamination using MycoAlert™ mycoplasma detection kit (Lonza, LT07-318 and LT07-518), suggesting no contamination.
Commonly misidentified lines (See ICLAC register)	N/A

Animals and other organisms

Policy information about studies involving animals; ARRIVE guidelines recommended for reporting animal research

Laboratory animals	NOD/ShiLtSz-scid/IL2Rgnull (NOD.Cg-PrkdcscidIl2rgtm1Wjl/SzJ or Nod scid gamma (NSG mice) for in vivo experiments.
Wild animals	not used
Field-collected samples	not used
Ethics oversight	All experiments were performed with authorization from the Institutional Review Board for Human Research at the CHA University (1044308-202204-LR-023-02) to use human ESC cell line cells. All animal protocols were reviewed and approved by the Institutional Animal Care and Use Committees of the CHA University (IACUC220090), and all animal procedures were performed in accordance with approved guidelines and regulations.

Note that full information on the approval of the study protocol must also be provided in the manuscript.

Flow Cytometry

Plots

Confirm that:

☒ The axis labels state the marker and fluorochrome used (e.g. CD4-FITC).

☒ The axis scales are clearly visible. Include numbers along axes only for bottom left plot of group (a 'group' is an analysis of identical markers).

☒ All plots are contour plots with outliers or pseudocolor plots.

☒ A numerical value for number of cells or percentage (with statistics) is provided.

Methodology

Sample preparation	Single cell suspensions were prepared from the cells. After removing red blood cells, cells were stained with MACS buffer at 4 °C in the presence of Fc Block (BD Biosciences 553141, dilution 1:100). After washing several times with PBS, stained cells were resuspended in PBS and analyzed by flow cytometry .
Instrument	Data was collected using Accuri C6 Plus flow cytometer equipped with BD Accuri C6 plus (BD Biosciences). Magnetic-activated cell sorting (MACS) using the CD34 MicroBead Kit (130-046-703, Miltenyi Biotec) was performed according to the manufacturer's instructions.
Software	Collection was performed using analysis usig FlowJo software or Accuri C6 software
Cell population abundance	Reanalysis of post-sort fractions in CD34+ cells>95%
Gating strategy	As per gating strategy figure

☒ Tick this box to confirm that a figure exemplifying the gating strategy is provided in the Supplementary Information.
