# Supplementary material for: Ferric citrate and apo-transferrin enable erythroblast maturation with β-globin from hemogenic endothelium
Source: NPJ Regen Med. 2023 Aug 25;8:46. doi: 10.1038/s41536-023-00320-4 (PMC10457393; doi:10.1038/s41536-023-00320-4)
Supplement: Supplementary file 2 — Supplementary data [file 41536_2023_320_MOESM2_ESM.pdf]

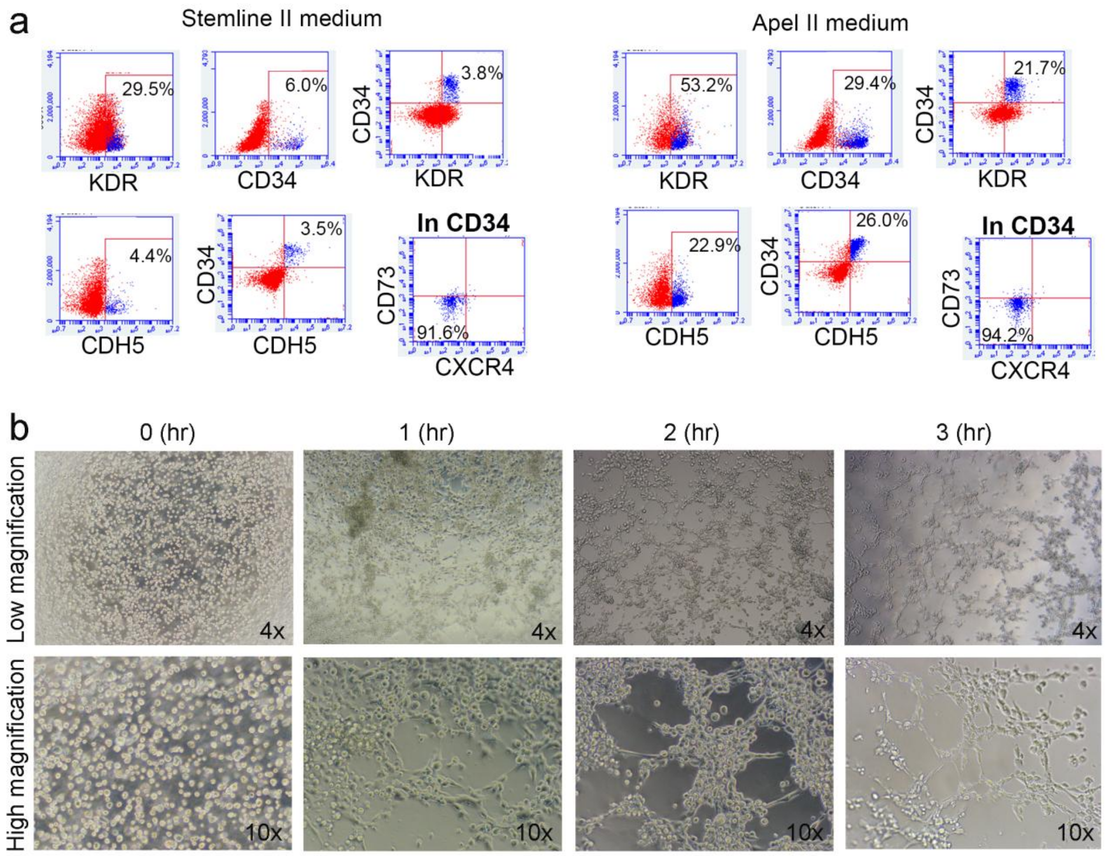

Supplementary Figure 1. Comparison of Apel-II and Stemline II medium with respect to the generation of HE. (a) Flow cytometry data for KDR, CD34, and CDH5 cells in differentiated HE on day six. The percentages of KDR, CD34, and CDH5 cells were increased in the Apel-II medium compared to those in the Stemline II medium. The frequency of $CD34^{+}KDR^{+}$ cells and $CD34^{+}CHD5^{+}$ cells was higher in the Apel-II medium than that in the Stemline II medium, with an increase of approximately 5.7- and 7.4-fold, respectively, in the frequency. No differences were observed between the two media with respect to the percentages of $CD34^{+}CXCR4^{-}CD73^{-}$ definitive HE cells. (b) Images of tube formation assay. The hESC-derived $CD34^{+}$ HEs were cultured in Matrigel-coated 24-well plates with EGM media for 3 hours. Magnification 4x, 10x.

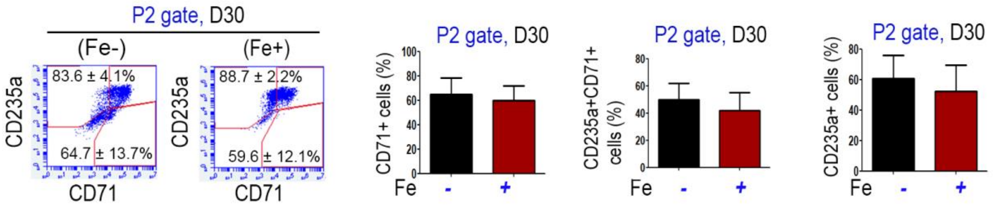

Supplementary Figure 2. Markers for RBC were expressed in immature (P2) erythroblasts. Regardless of Fe treatment, CD235a+CD71+ cells were enriched in immature (P2) erythroblasts population. The CD235a+ and CD235a+CD71+ cells were increased, compared that of CD71+ only phenotype cells at day 30, suggesting maturation of erythroid lineage cells. n = 4–5 per group.

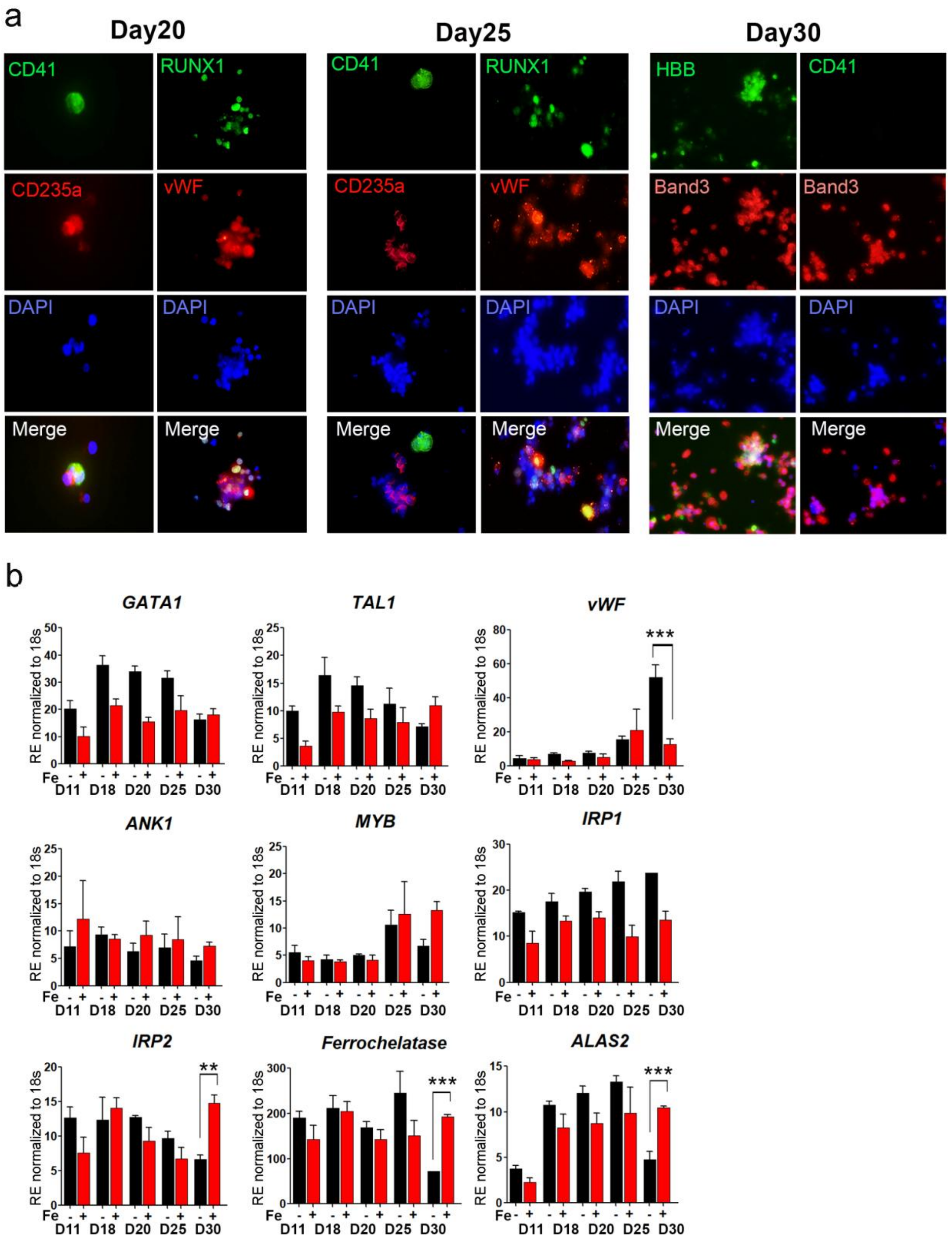

Supplementary Figure 3. The markers for differentiated cells including megakaryocyte erythrocyte progenitors- erythroid lineage cells were investigated according to differentiation. (a) Immunostaining data clearly showed that Runx1, vWF, CD235a, and CD41 were detected in megakaryocyte erythrocyte progenitors. At day30, Band3 and β-globin were expressed in differentiated cells, but not CD41. Magnification x 40. **(b)** The results of qRT-PCR revealed a significantly high mRNA expression corresponding to *GATA1, TAL1, vWF, ANK1, MYB, IRP1, IRP2, ferrochelatase, and ALAS2* in differentiated cells in the cells. Data are represented as means ± SEM derived from three independent experiments performed with duplicates. n = 3 per group.

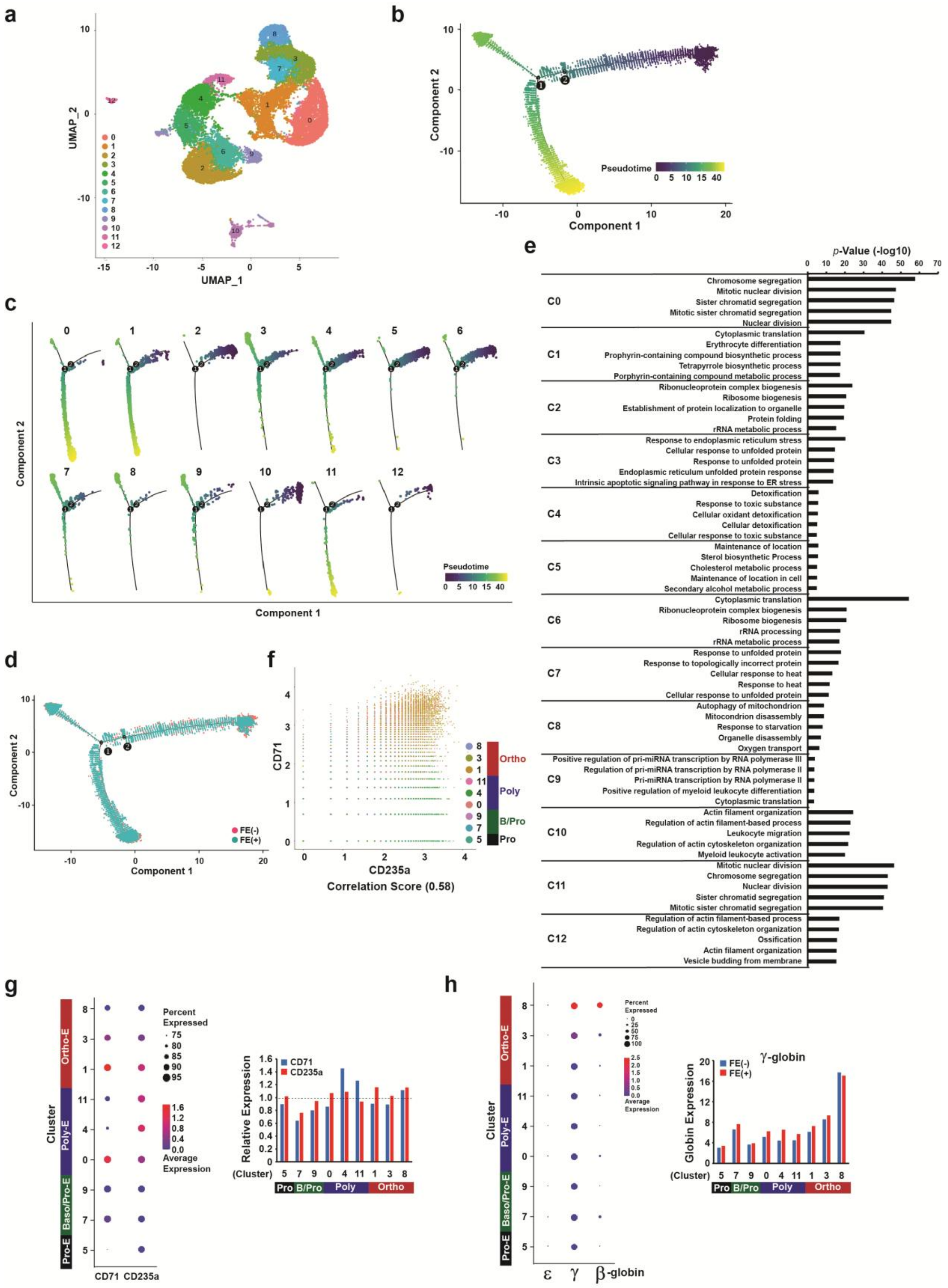

Supplementary Figure 4. Enrichment of functional transcripts in β-globin^{+} cells was demonstrated by single-cell RNA sequencing. (a) UMAP visualization of heterogenous clusters of hPSC-derived erythrocytes (total, *n*=19197; cells derived from control=8,785;

cells treated with Fe=10,412); 13 clusters were identified based on expressed UMIs. **(b)** Cell differentiation trajectory with development pseudo-time of derived erythrocytes evaluated by Monocle2. **(c)** Cell differentiation trajectory of each cluster. Each dot represents a single cell, and each color indicates pseudotime. **(d)** Comparison of cell differentiation trajectory between Fe treated- and control erythrocytes. **(e)** Enriched GO terms and *P* values of each cluster. **(f)** Scatterplots showing the expression of representative markers (CD235a and CD71) in erythrocyte development. Cells from different clusters are represented by indicated colors. **(g)** Dot plot analysis for the expression of erythrocyte markers (CD235a and CD71) in each cluster. The right panel indicates the relative expression of CD235a and CD71 in Fe-treated erythrocytes compared to that in the control. **(h)** Dot plot analysis for ε-, χ-, and β-globin expression in each cluster. The right panel presents the expression level of genes encoding χ-globin.

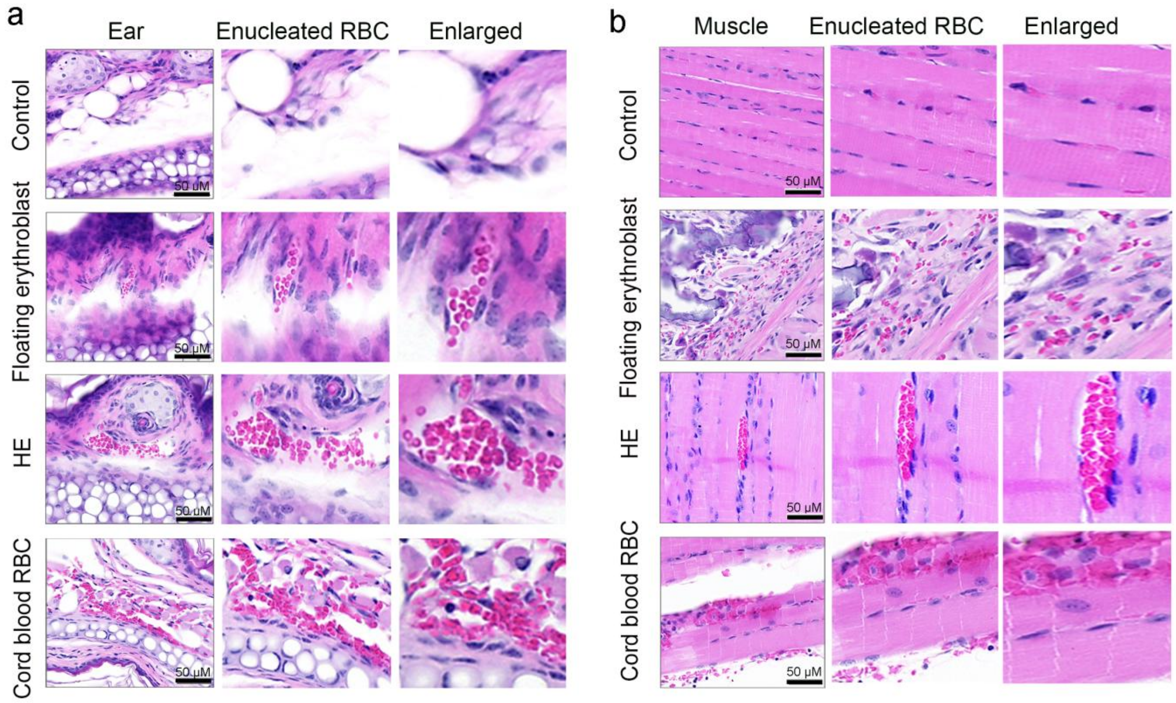

Supplementary Figure 5. H&E staining of floating erythroblasts, CB-RBC, and HE in the ear skin and muscles of NSG mice. (a) Immunostaining showed mature RBCs in the ear skin of NSG mice. Mice were euthanized three days after cell injection, and the ear skin tissue was analyzed via H&E staining. Scale bar = 50 μM. **(b)** Immunostaining showed biconcave disk-shaped RBCs in the muscle of NSG mice, suggesting maturation of erythroblasts in vivo. Scale bar = 50 μM.

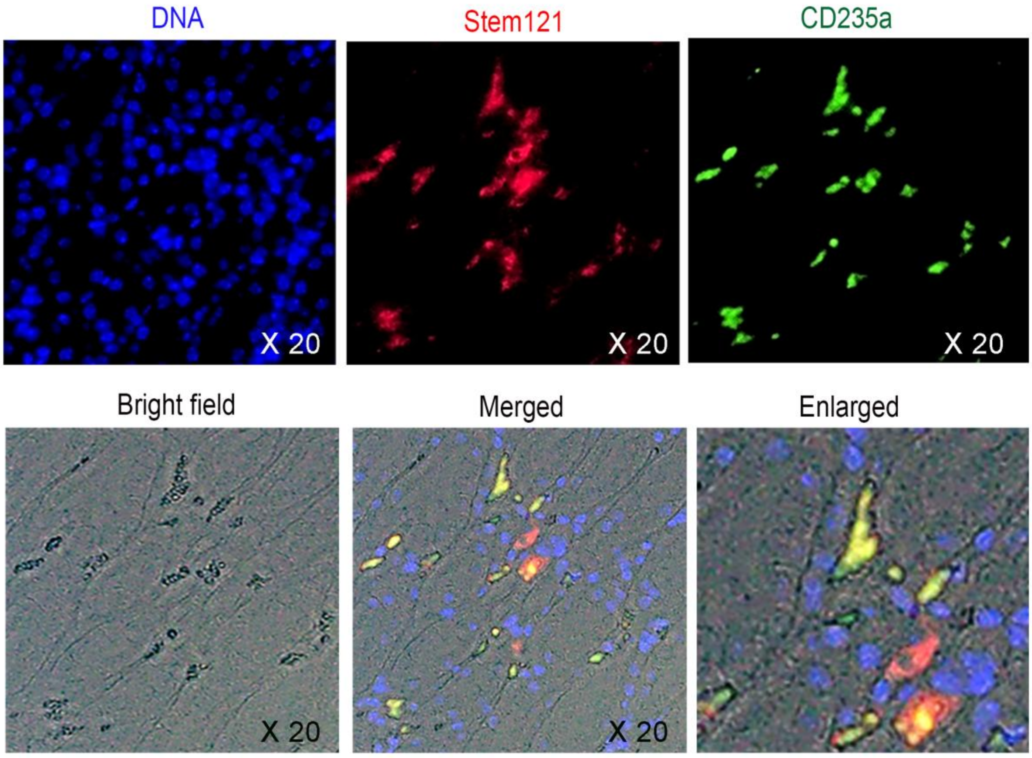

Supplementary Figure 6. Immunohistochemical analysis of floating erythroblasts in the muscles of NSG mice. Co-expression of human-specific markers stem121 and CD235a was observed in biconcave disk-shaped human RBCs derived from the muscles, suggesting RBC maturation. Magnification 20 x.

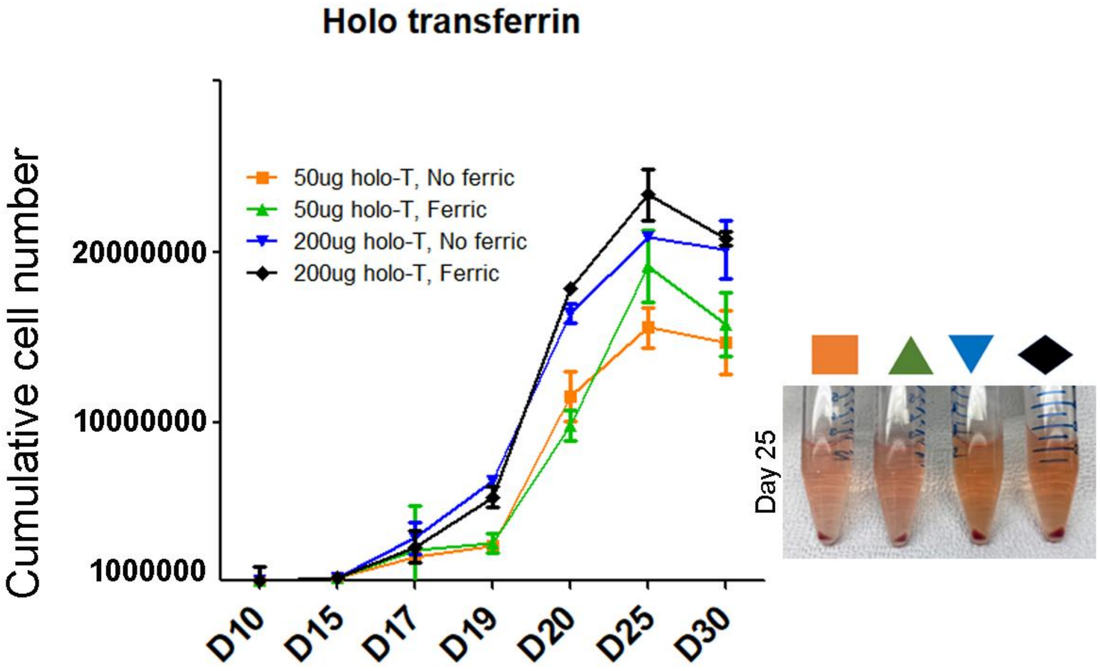

Supplementary Figure 7. The cumulative numbers of erythroblasts were increased by holo-transferrin. Regardless of ferric citrate adding, the cumulative numbers of erythroblasts in vitro culture were increased according to holo-transferrin concentration. Cumulated cell numbers till 30 days will be measured at least 2 independent experiments. (n=2, SD value)
